# Old Lineage on an Old Island: *Pixibinthus*, a New Cricket Genus Endemic to New Caledonia Shed Light on Gryllid Diversification in a Hotspot of Biodiversity

**DOI:** 10.1371/journal.pone.0150920

**Published:** 2016-03-30

**Authors:** Jérémy Anso, Laure Barrabé, Laure Desutter-Grandcolas, Hervé Jourdan, Philippe Grandcolas, Jiajia Dong, Tony Robillard

**Affiliations:** 1 Muséum national d'Histoire naturelle, Institut de Systématique, Évolution, Biodiversité, ISYEB - UMR 7205 – CNRS, MNHN, UPMC, EPHE, Sorbonne Universités, Paris, France; 2 Institut Méditerranéen de Biodiversité et d’Écologie marine et continentale (IMBE), Aix Marseille Université, CNRS, IRD, Avignon Université UMR IRD 237 IMBE, Centre IRD Nouméa, Nouvelle-Calédonie; 3 Laboratoire de Botanique et d’Ecologie Végétales Appliquées, Herbarium NOU, UMR 123: botAnique et Modélisation de l’Architecture des Plantes et des vegetations (AMAP), Centre IRD Nouméa, Nouvelle-Calédonie; Consiglio Nazionale delle Ricerche (CNR), ITALY

## Abstract

Few studies have focused on the early colonization of New Caledonia by insects, after the re-emergence of the main island, 37 Myr ago. Here we investigate the mode and tempo of evolution of a new endemic cricket genus, *Pixibinthus*, recently discovered in southern New Caledonia. First we formally describe this new monotypic genus found exclusively in the open shrubby vegetation on metalliferous soils, named ‘maquis minier’, unique to New Caledonia. We then reconstruct a dated molecular phylogeny based on five mitochondrial and four nuclear loci in order to establish relationships of *Pixibinthus* within Eneopterinae crickets. *Pixibinthus* is recovered as thesister clade of the endemic genus *Agnotecous*, mostly rainforest-dwellers. Dating results show that the island colonization by their common ancestor occurred around 34.7 Myr, shortly after New Caledonia re-emergence. *Pixibinthus* and *Agnotecous* are then one of the oldest insect lineages documented so far for New Caledonia. This discovery highlights for the first time two clear-cut ecological specializations between sister clades, as *Agnotecous* is mainly found in rainforests with 19 species, whereas *Pixibinthus* is found in open habitats with a single documented species. The preference of *Pixibinthus* for open habitats and of *Agnotecous* for forest habitats nicely fits an acoustic specialization, either explained by differences in body size or in acoustic properties of their respective habitats. We hypothesize that landscape dynamics, linked to major past climatic events and recent change in fire regimes are possible causes for both present-day low diversity and rarity in genus *Pixibinthus*. The unique evolutionary history of this old New Caledonian lineage stresses the importance to increase our knowledge on the faunal biodiversity of ‘maquis minier’, in order to better understand the origin and past dynamics of New Caledonian biota.

## Introduction

Understanding the origin of island biota requires that the evolutionary history of clades is reconstructed in details and linked to the evolution of local environments [1,2]. This task is both especially interesting and demanding in hotspots of biodiversity, such as New Caledonia in the Southwest Pacific, where richness and endemism reach outstanding levels [3,4,5]. New Caledonia has a long and complex environmental history since the main island re-emerged 37 ± 2 Myr ago [6,7], after having been largely covered with an ophiolithic layer during its obduction under the Pacific plate [7,8]. This layer has been progressively reduced to only one third of the island surface after several intense weathering events under climate control. These geological and climatic events led to the establishment of a mosaic of soils, including metalliferous soils (rich in heavy metals such as magnesium, manganese and nickel), habitats and landscapes (e.g., [9]). The present-day vegetation on metalliferous soils is constituted by two main native types, rainforest and open shrubby vegetation named ‘maquis minier’, both harboring many endemic species [10,11]. Many studies refer to the interaction between metalliferous soils and the corresponding vegetation as a peculiar and original system dating back to the first ages of the island colonization. Pre-adaptation or adaptation to these specific soils on ultramafic rocks would have been an early engine of diversification of endemism for at least a major part of New Caledonian biota with the subsequent prediction that island old groups have narrow relationships to metalliferous soils [10,12,13]. This theory has both been corroborated and refuted depending on group of organism studied (e.g., [14,15,16,17]) and a dated phylogenetic framework is now clearly required to clarify this subject. The present-day vegetation dynamics can also obscure the reconstruction of past processes. The balance between ‘maquis minier’ and forest is controlled by climatic variations, fire regimes and human influences leading to a present increase and over-representation of this open vegetation type [18,19,20]. Therefore, ‘maquis minier’ is often seen as a secondary and disturbed vegetation type when it is actually a rich original endemic formation potentially indicative of early New Caledonian ecosystems [21,22].

Our study is aimed at unraveling the evolution of an insect lineage endemic to ‘maquis minier’, to date its origin, to emphasize on its adaptation to the habitat and to suggest future research directions for better understanding its relationships to landscape dynamics and human disturbance. We focused on the insect group of crickets (Orthoptera, Grylloidea) that gained increasing attention from the scientific community as they both play a significant role in ecosystem functioning [23]and are relatively sensitive to environmental disturbances [24]. In New Caledonia, the cricket fauna is rich and has been recently studied, providing some insights on the dynamics of the micro-endemism and of the adaptation to the environment [25,26,27,28]. This fauna was still insufficiently known in ‘maquis minier’ where we concentrated our efforts, permitting the discovery and description of a new genus, *Pixibinthus*Robillard and Anso,gen. nov., that was used to ask the present questions.

## Material and Methods

### Study sites and cricket sampling

Intensive cricket samplings were performed from October 2013 to March 2014 to study the composition of cricket species along a gradient from ‘maquis minier’ to rainforest (S3 Table). We selected our study sites on the uniform metalliferous soil located in the southern part of Grande Terre. We subsequently recorded for each sampling site the absence / presence of each cricket species. For each cricket sampled in the field, time of day and habitats were noted. Crickets were identified as morphospecies at the Institut de Recherche pour le Développement (IRD) in Noumea, and identifications were checked by comparisons with the collections of the Muséum national d’Histoire naturelle, Paris (MNHN). Specimens were deposited in the collections of the MNHN, and in collections of the IRD, Noumea (ONNC), and in collection of the Institut Agronomique néo-Calédonien, Pocquereux (CXMNC). Field Permit: the Province Sud Environment Office (DENV- Direction de l’Environnement de la Province Sud).

### Taxon description

Morphological descriptions follow the pattern and terminology of recent publications (e.g., [29,30]). Direct observations and dissections have been made using a binocular microscope Leica MZ16 at magnifications up to 115×. Male tegminal veins and cells follow terminology of Ragge [31] and Robillard & Desutter-Grandcolas [32]. Male and female genitalia have been dissected in softened or fresh specimens. Male genitalia were dissected by cutting the membranes between the paraprocts and the subgenital plate; in females, copulatory papilla were dissected by cutting the membranes between the ovipositor and the subgenital plate. Genitalia have been cleared with cold KOH and preserved in glycerine in vials pinned under specimens. Male genitalia terminology follows Desutter [33], modified in Desutter-Grandcolas [34] and Robillard & Desutter-Grandcolas [32]. Photographs of male genitalia were obtained using an AmScope MU1000 digital camera (www.Amscope.com). Genitalia were stained with a drop of Punktol (JLB, Germany).

### Nomenclatural Acts

The electronic edition of this article conforms to the requirements of the amended International Code of Zoological Nomenclature, and hence the new names contained herein are available under that Code from the electronic edition of this article. This published work and the nomenclatural acts it contains have been registered in ZooBank, the online registration system for the ICZN. The ZooBank LSIDs (Life Science Identifiers) can be resolved and the associated information viewed through any standard web browser by appending the LSID to the prefix “http://zoobank.org/”. The LSID for this publication is: urn:lsid:zoobank.org:pub: AFAE2A4F-7161-4100-A1DB-D420FA5C0435. The electronic edition of this work was published in a journal with an ISSN, and has been archived and is available from the following digital repositories: PubMed Central, LOCKSS.

### Bioacoustic study

Collected crickets from the field were recorded with a modified condenser microphone (CM16 Avisoft Bioacoustics, Berlin, frequency range: 3–150 kHz ± 6 dB, R. Specht pers. comm.) in a sound attenuated room controlled for temperature in the MNHN. The insects were placed over night alone in a cage made of mosquito net to avoid echoes. The microphone was placed 30 cm from the cage. Automatic recordings were made using the program Avisoft Triggering Harddisk Recorder version 2.97 and a 8-Pre MOTU sound card at a sampling frequency of 96 kilo-samples per second (16 bit). The temporal acoustic activity of crickets was obtained in the field by ambient acoustic recordings with a SongMeter SM2 Bat sensor (Wildlife Acoustics, concord, NY, U.S.A.) at Pic du Grand Kaori during 72 hours.

Cricket song terminology follows Ragge & Reynolds [35]: one song unit is called a syllable and corresponds to one opening-closing cycle of the male forewings; a group of syllables forming a cricket call is named an echeme. We used the computer software Avisoft-SASLab pro V. 5.2.06 (Avisoft bioacoustics; Specht 2012) to automatically analyze cricket calls. We used automatic analyses to calculate syllable duration and period, number of syllable per echeme, duty-cycle, and dominant frequency. Eight calling songs from 4 different males were used for the analysis (two per male). Measurements acquired from automatic analyses were verified manually in order to avoid errors or aberrant values. Sound tracks were deposited in the Sound Library of the MNHN, Paris (MNHN-SO-2015-9, 10, 11, 12, 13, 14).

### Abbreviations

General morphology: FW: forewing; Tarsomere III-1: basal segment of hind leg tarsomere; T: tibiae.

Male genitalia: ect ap: ectophallic apodeme; ect arc: ectophallic arc; ect f: ectophallic fold; ect lat exp: ectophallic lateral expansion; end ap: endophallic apodeme; end s: endophallic sclerite; pse p: pseudepiphallic paramere.

Tegminal venation: 1A-4A: first to fourth anal veins; CuA: anterior cubitus; CuP: posterior cubitus; M: median vein; Sc: subcostal vein; R: radial vein; di: diagonal vein; ob: oblique veins, c1-3: first to third cells of C alignment; d1 cell (mirror): first cell(s) of D alignment; d2: second cell of D alignment; e1: first cell of E alignment.

Morphological measurements: FIIIL: length of hind femora; FIIIW: width of hind femora; FWL: forewing length; FWW: forewing width (at the level of maximal width); Ias: inner spines on TIII dorsal side, above the spurs; Ibs: inner spines on TIII dorsal side, between the spurs; Oas: outer spines on TIII dorsal side, above the spurs; Obs: outer spines on TIII dorsal side, between the spurs; OL: ovipositor length; PronL: pronotum length; PronW: pronotum width; TIIIL: length of hind tibiae; TaIIIs: spines on outer edge of third hind tarsomere, not including the apical spine.

Acoustics: fd: dominant frequency; TSR: tooth-strike rate.

### Molecular phylogeny

#### Taxon sampling

Two molecular datasets were built aiming both to investigate the phylogenetic placement of *Pixibinthus* within crickets (= dataset 1), and to generate a molecular dating to estimate divergence times of *Pixibinthus* and its close relatives (= dataset 2).

The molecular sampling of dataset 1 comprised six individuals of *Pixibinthus* (from three different locations). As *Pixibinthus* shares the typical morphology of the Lebinthini tribe within the subfamily Eneopterinae (see below), a special emphasis has been put on the taxonomical sampling of this tribe, by incorporating representatives of the genera *Agnotecous* Saussure, 1878 (nine species; 14 samples), *Lebinthus* Stål, 1877 (six species; seven samples), *Cardiodactylus* Saussure, 1878 (four species; five samples), and *Swezwilderia* Chopard, 1929 (one species). The sampling was then completed with other species belonging to the other four current tribes of the Eneopterinae subfamily (sensu [36]): Eurepini (one species), Eneopterini (one species), Nisitrini (two species; three samples), and Xenogryllini (one species). A total of 26 species were then used in the ingroup sampling of dataset 1 (corresponding to 39 samples; Tables 1 and 2). Two species from a different cricket subfamily (Gryllinae: *Acheta domesticus* (Linnaeus, 1758) and *Gryllus bimaculatus* De Geer, 1773) were finally used as most external outgroups.

**Table 1:** List of taxa investigated in the molecular study, with voucher information, and country and locality origin of samples, taxonomical classification. molecular dataset. The phylogeny-based taxonomies of Chintauan-Marquier et al. [37] for Gryllidea and Robillard & Desutter-Grandcolas [36] for Eneopterinae were used; superfamilies and families are indicated for all taxa, and subfamilies and tribes for ingroup taxa only. Abbreviations used for subfamilies: ENEO = Eneopterinae, GRYL = Gryllinae; Tribes: Ene = Eneopterini, Eur = Eurepini, Gry = Gryllini, Leb = Lebinthini, Nis = Nisitrini, Xen = Xenogryllini. N/A corresponds to non-available data.

Species	Superfamily / Family	Ingroup: subfamily / tribe	Country	Locality	Vouchers
*Acheta domesticus* (Linnaeus, 1758)	GRYLLOIDEA / Gryllidae	GRYL / Gry	France	Laboratory strain	MNHN-EO-ENSIF3523
*Afrophaloria amani* Desutter-Grandcolas, 2015	GRYLLOIDEA / Phalangopsidae		Tanzania	Amani	MNHN-EO-ENSIF3341
*Agnotecous albifrons* Desutter-Grandcolas, 1997 CT	GRYLLOIDEA / Gryllidae	ENEO/ Leb	New Caledonia	Col Toma	MNHN-EO-ENSIF2767
*Agnotecous albifrons* Desutter-Grandcolas, 1997 Ge	GRYLLOIDEA / Gryllidae	ENEO/ Leb	New Caledonia	Gelima	MNHN-EO-ENSIF1771
*Agnotecous azurensis* Desutter-Grancolas, 2006 GK	GRYLLOIDEA / Gryllidae	ENEO/ Leb	New Caledonia	Grand Kaori	MNHN-EO-ENSIF2777
*Agnotecous azurensis* Desutter-Grancolas, 2006 RB	GRYLLOIDEA / Gryllidae	ENEO/ Leb	New Caledonia	Rivière Bleue (Pourina)	MNHN-EO-ENSIF2780
*Agnotecous clarus* Desutter-Grandcolas, 2006	GRYLLOIDEA / Gryllidae	ENEO/ Leb	New Caledonia	Pic du Pin	MNHN-EO-ENSIF2788
*Agnotecous meridionalis* Desutter-Grandcolas, 2006 IDP	GRYLLOIDEA / Gryllidae	ENEO/ Leb	New Caledonia	Ile des Pins	MNHN-EO-ENSIF2772
*Agnotecous meridionalis* Desutter-Grandcolas, 2006 PB	GRYLLOIDEA / Gryllidae	ENEO/ Leb	New Caledonia	Port Boisé	MNHN-EO-ENSIF2771
*Agnotecous obscurus* (Chopard, 1970) Ao	GRYLLOIDEA / Gryllidae	ENEO/ Leb	New Caledonia	Aoupinié	MNHN-EO-ENSIF2786
*Agnotecous obscurus* (Chopard, 1970) Ma	GRYLLOIDEA / Gryllidae	ENEO/ Leb	New Caledonia	Mandjélia	MNHN-EO-ENSIF2785
*Agnotecous occidentalis* Desutter-Grandcolas, 2006	GRYLLOIDEA / Gryllidae	ENEO/ Leb	New Caledonia	Col des Roussettes	MNHN-EO-ENSIF2765
*Agnotecous robustus* (Chopard, 1915)	GRYLLOIDEA / Gryllidae	ENEO/ Leb	New Caledonia	Aoupinié	MNHN-EO-ENSIF2752
*Agnotecous sarramea* Desutter-Grancolas, 1997	GRYLLOIDEA / Gryllidae	ENEO/ Leb	New Caledonia	Mé Aréto	MNHN-EO-ENSIF2764
*Agnotecous yahoue* Otte, 1987 Ko	GRYLLOIDEA / Gryllidae	ENEO/ Leb	New Caledonia	Monts Koghis	MNHN-EO-ENSIF2766
*Agnotecous yahoue* Otte, 1987 Mo	GRYLLOIDEA / Gryllidae	ENEO/ Leb	New Caledonia	Mont Mou	MNHN-EO-ENSIF2773
*Anaxipha* sp. affinis *nitida* (Chopard, 1925)	GRYLLOIDEA / Trigonidiidae		French Guiana	Arataye	MNHN-EO-ENSIF3260
*Bullita* sp.	GRYLLOIDEA / Trigonidiidae		New Caledonia	Yahoue	MNHN-EO-ENSIF3393
*Cardiodactylus enkraussi* Otte, 2007	GRYLLOIDEA / Gryllidae	ENEO/ Leb	Vanuatu	Espiritu Santo, Vathé	MNHN-EO-ENSIF2366
*Cardiodactylus guttulus* (Matsumura, 1913)	GRYLLOIDEA / Gryllidae	ENEO/ Leb	Japan		MNHN-EO-ENSIF1193
*Cardiodactylus novaeguineae* (Haan, 1842)	GRYLLOIDEA / Gryllidae	ENEO/ Leb	Vanuatu	Espiritu Santo, Peavot	MNHN-EO-ENSIF2030
*Cardiodactylus novaeguineae* (Haan, 1842) NC	GRYLLOIDEA / Gryllidae	ENEO/ Leb	New Caledonia	Lifou	MNHN-EO-ENSIF1921
*Cardiodactylus tankara* Robillard, 2009	GRYLLOIDEA / Gryllidae	ENEO/ Leb	Vanuatu	Espiritu Santo, Butmas	MNHN-EO-ENSIF2410
*Cearacesa* sp.	GRYLLOIDEA / Gryllidae		Brazil	Pernambuco	MNHN-EO-ENSIF3270
*Diatrypa* sp.	GRYLLOIDEA / Gryllidae		French Guiana	Arataye	MNHN-EO-ENSIF3261
*Ectecous* sp.	GRYLLOIDEA / Phalangopsidae		French Guiana	Arataye	MNHN-EO-ENSIF3384
*Eneoptera guyanensis* Chopard, 1931	GRYLLOIDEA / Gryllidae	ENEO / Ene	French Guiana	Montagne de Kaw	MNHN-EO-ENSIF2741^1^ / MNHN-EO-ENSIF3687^2^
Eurepini sp.	GRYLLOIDEA / Gryllidae	ENEO / Eur	Australia	Northern Territory, Litchfield National Park	MNHN-EO-ENSIF3155
*Fryerius* sp.	GRYLLOIDEA / Gryllidae		Comoros	Moheli	MNHN-EO-ENSIF3378
*Gryllotalpa* sp.	GRYLLOTALPOIDEA / Gryllotalpidae		Mozambique	Cabo Delgado	MNHN-EO-ENSIF3315
*Gryllus bimaculatus* De Geer, 1773	GRYLLOIDEA / Gryllidae	GRYL / Gry	France	Laboratory strain	MNHN-EO-ENSIF3524/3404
*Homeogryllus orientalis* Desutter, 1985	GRYLLOIDEA / Phalangopsidae		Mozambique	Cabo Delgado	MNHN-EO-ENSIF3603
*Lebinthus lifouensis* Desutter-Grandcolas, 1997	GRYLLOIDEA / Gryllidae	ENEO/ Leb	New Caledonia	Lifou	MNHN-EO-ENSIF1346
*Lebinthus luae* Robillard & Tan, 2013	GRYLLOIDEA / Gryllidae	ENEO/ Leb	Singapore	Labrador park	MNHN-EO-ENSIF2740
*Lebinthus nattawa* Robillard, 2009	GRYLLOIDEA / Gryllidae	ENEO/ Leb	Vanuatu	Nattawa, Santo	MNHN-EO-ENSIF2564
*Lebinthus santoensis* Robillard, 2009 P	GRYLLOIDEA / Gryllidae	ENEO/ Leb	Vanuatu	Espiritu Santo, Peavot	MNHN-EO-ENSIF2484
*Lebinthus santoensis* Robillard, 2009 V	GRYLLOIDEA / Gryllidae	ENEO/ Leb	Vanuatu	Espiritu Santo, Vathé	MNHN-EO-ENSIF2437
*Lebinthus* sp. PNG	GRYLLOIDEA / Gryllidae	ENEO/ Leb	Papua New Guineae	New Ireland	MNHN-EO-ENSIF117^1^ / MNHN-EO-ENSIF157^2^
*Lebinthus villemantae* Robillard, 2010	GRYLLOIDEA / Gryllidae	ENEO/ Leb	Indonesia	Sulawesi, Bulu Saraun	MNHN-EO-ENSIF2739
*Luzaridella obscura* Desutter-Grandcolas, 1992	GRYLLOIDEA / Phalangopsidae		French Guiana	Arataye	MNHN-EO-ENSIF3253
*Microlandreva* sp. Chopard, 1958	GRYLLOIDEA / Gryllidae		Mayotte		MNHN-EO-ENSIF3307
*Nisitrus vittatus* (Haan, 1842) Se	GRYLLOIDEA / Gryllidae	ENEO / Nis	Malaysia	Selangor, Mount Kira	MNHN-EO-ENSIF3134
*Nisitrus vittatus* (Haan, 1842) Si	GRYLLOIDEA / Gryllidae	ENEO / Nis	Singapore	Bukit Timah Natural Reserve	MNHN-EO-ENSIF2742
*Ornebius xanthopterus* Guérin-Méneville, 1844	GRYLLOIDEA / Mogoplistidae		Mauritius	Le morne	Collection SH-2011-016
*Paranisitra longipes* Chopard, 1925	GRYLLOIDEA / Gryllidae	ENEO / Nis	Philippines	Luzon, Mount Makiling	MNHN-EO-ENSIF3157
*Pixibinthus sonicus* Anso & Robillard, sp. nov. FN1	GRYLLOIDEA / Gryllidae	ENEO/ Leb	New Caledonia	Forêt Nord	MNHN-EO-ENSIF99
*Pixibinthus sonicus* Anso & Robillard, sp. nov. FN2	GRYLLOIDEA / Gryllidae	ENEO/ Leb	New Caledonia	Forêt Nord	MNHN-EO-ENSIF83
*Pixibinthus sonicus* Anso & Robillard, sp. nov. GK1	GRYLLOIDEA / Gryllidae	ENEO/ Leb	New Caledonia	Grand Kaori	MNHN-EO-ENSIF150
*Pixibinthus sonicus* Anso & Robillard, sp. nov. GK2	GRYLLOIDEA / Gryllidae	ENEO/ Leb	New Caledonia	Grand Kaori	MNHN-EO-ENSIF125
*Pixibinthus sonicus* Anso & Robillard, sp. nov. RB1	GRYLLOIDEA / Gryllidae	ENEO/ Leb	New Caledonia	Rivière Bleue (Rivière Blanche)	MNHN-EO-ENSIF99
*Pixibinthus sonicus* Anso & Robillard, sp. nov. RB2	GRYLLOIDEA / Gryllidae	ENEO/ Leb	New Caledonia	Rivière Bleue (Rivière Blanche)	MNHN-EO-ENSIF133
*Pteroplistes masinagudi* Jaiswara, 2014	GRYLLOIDEA *incertae sedis*		India	Tamil Nadu	Collection BNHS
*Swezwilderia* sp.	GRYLLOIDEA / Gryllidae	ENEO /?	Fiji	Viti Levu	MNHN-EO-ENSIF2737
*Xenogryllus marmoratus* Bolívar, 1890	GRYLLOIDEA / Gryllidae	ENEO / Xen	Japan	Honshu, Nara City	MNHN-EO-ENSIF3161

**Table 2:** Genbank accession numbers of studied taxa. Newly generated sequences are indicated with an asterisk (and would be very soon referred to a genbank accession number). The superscript numbers refer to the publication where the sequences were first published: ^1^[27], ^2^[38], ^3^[39], ^4^[40], ^5^[41], ^6^[42], ^7^[43], ^8^[37], ^9^[44].

Species	dataset 1	dataset 2	16S	12S	Cytb	CO1	CO2	28SA	EF1a	H3	18S
*Acheta domesticus* (Linnaeus, 1758)	x	x	AF248698^2^	Z97611^9^	AF248682^2^	JX897403^1^	JX897439^1^	JX897465^1^	GQ886692^3^	KR903150*	AD18SITS1*
*Afrophaloria amani* Desutter-Grandcolas, 2015		x	KR903696^8^	KR903858^8^	KR903353^8^	N/A	N/A	KR903524^8^	N/A	KR903173^8^	KR904049^8^
*Agnotecous albifrons* Desutter-Grandcolas, 1997 CT	x	x	JX897353^1^	JX897394^1^	JX897314^1^	JX897418^1^	JX897446^1^	JX897490^1^	JX897527^1^	JX897572^1^	JX897583^1^
*Agnotecous albifrons* Desutter-Grandcolas, 1997 Ge	x		JX897354^1^	JX897396^1^	JX897316^1^	JX897416^1^	JX897445^1^	N/A	JX897529^1^	JX897574^1^	JX897585^1^
*Agnotecous azurensis* Desutter-Grancolas, 2006 GK	x		JX897360^1^	JX897389^1^	JX897331^1^	JX897426^1^	N/A	JX897472^1^	JX897505^1^	JX897565^1^	JX897593^1^
*Agnotecous azurensis* Desutter-Grancolas, 2006 RB	x	x	JX897358^1^	JX897376^1^	JX897329^1^	JX897423^1^	JX897453^1^	JX897475^1^	JX897502^1^	JX897566^1^	JX897595^1^
*Agnotecous clarus* Desutter-Grandcolas, 2006	x	x	JX897347^1^	JX897400^1^	JX897324^1^	JX897407^1^	JX897451^1^	JX897492^1^	JX897523^1^	JX897554^1^	JX897590^1^
*Agnotecous meridionalis* Desutter-Grandcolas, 2006 IDP	x		JX897349^1^	JX897401^1^	JX897311^1^	JX897420^1^	N/A	JX897488^1^	JX897519^1^	JX897553^1^	JX897579^1^
*Agnotecous meridionalis* Desutter-Grandcolas, 2006 PB	x	x	JX897350^1^	JX897402^1^	JX897313^1^	JX897410^1^	JX897442^1^	JX897489^1^	JX897520^1^	JX897550^1^	JX897597^1^
*Agnotecous obscurus* (Chopard, 1970) Ao	x		JX897356^1^	JX897398^1^	JX897319^1^	JX897415^1^	JX897449^1^	N/A	JX897510^1^	N/A	JX897591^1^
*Agnotecous obscurus* (Chopard, 1970) Ma	x	x	JX897357^1^	JX897393^1^	JX897320^1^	JX897412^1^	JX897450^1^	JX897487^1^	JX897525^1^	JX897576^1^	JX897587^1^
*Agnotecous occidentalis* Desutter-Grandcolas, 2006	x	x	JX897362^1^	JX897386^1^	JX897322^1^	JX897434^1^	JX897461^1^	N/A	JX897512^1^	JX897570^1^	JX897589^1^
*Agnotecous robustus* (Chopard, 1915)	x	x	JX897359^1^	JX897375^1^	JX897333^1^	JX897406^1^	JX897443^1^	N/A	JX897498^1^	JX897555^1^	JX897588^1^
*Agnotecous sarramea* Desutter-Grancolas, 1997	x	x	JX897372^1^	JX897380^1^	JX897342^1^	JX897430^1^	JX897456^1^	JX897471^1^	JX897511^1^	JX897561^1^	JX897598^1^
*Agnotecous yahoue* Otte, 1987 Ko	x	x	JX897366^1^	JX897387^1^	JX897334^1^	JX897438^1^	JX897463^1^	JX897478^1^	JX897500^1^	JX897549^1^	JX897602^1^
*Agnotecous yahoue* Otte, 1987 Mo	x		JX897367^1^	JX897388^1^	JX897337^1^	JX897437^1^	JX897462^1^	JX897479^1^	JX897501^1^	N/A	N/A
*Anaxipha* sp. affinis *nitida* (Chopard, 1925)		x	KR903735^8^	KR903906^8^	KR903396^8^	N/A	N/A	KR903570^8^	N/A	KR903220^8^	KR904094^8^
*Bullita* sp.		x	KR903810^8^	KR904001^8^	KR903473^8^	N/A	N/A	KR903649^8^	N/A	KR903307^8^	KR904183^8^
*Cardiodactylus enkraussi* Otte, 2007	x	x	JF972518 / JF972486^4^	JF972502^4^	N/A	KU705561*	KU705550*	N/A	KU705611*	KU705595*	JF972533^4^
*Cardiodactylus guttulus* (Matsumura, 1913)	x	x	JF972519^4^	JF972503^4^	JF972487^4^	KU705562*	N/A	KU705580*	KU705612*	KU705596*	JF972534^4^
*Cardiodactylus novaeguineae* (Haan, 1842)	x	x	JF972521^4^	JF972506^4^	JF972490^4^	KU705563*	KU705551*	KR903500*	KU705613*	KR903151*	JF972537^4^
*Cardiodactylus novaeguineae* (Haan, 1842) NC	x	x	AY905299^5^	AY905270^5^	AY905353^5^	KU705564*	KU705552*	KU705588*	KU705614*	KU705597*	AY905329^5^
*Cardiodactylus tankara* Robillard, 2009	x	x	JF972522^4^	JF972508^4^	JF972491^4^	KU715286*	KU726590*	KU705581*	N/A	KU705598*	JF972538^4^
*Cearacesa* sp.		x	KR903798^8^	KR903985^8^	KR903459^8^	N/A	N/A	KR903638^8^	N/A	KR903294^8^	KR904170^8^
*Diatrypa* sp.		x	KR903728^8^	KR903899^8^	KR903389^8^	N/A	N/A	KR903564^8^	N/A	KR903214^8^	KR904087^8^
*Ectecous* sp.		x	KR903726^8^	KR903897^8^	KR903387^8^	N/A	N/A	KR903562^8^	N/A	KR903212^8^	KR904085^8^
*Eneoptera guyanensis* Chopard, 1931	x	x	AY905301^5^	AY905272^5^	AY905355^5^	JX897404^1^	KU705553*	KU705582*	JX897495^1^	JX897547^1^	AY905331^5^
Eurepini sp.	x	x	KR903674*	KR903834*	KR903331*	KU705565*	KU705554*	KR903503*	N/A	KR903153*	KR904028*
*Fryerius* sp.		x	KR903730^8^	KR903901^8^	KR903391^8^	N/A	N/A	KR903566^8^	N/A	KR903216^8^	KR904089^8^
*Gryllotalpa* sp.		x	KR903783^8^	KR903961^8^	KR903443^8^	N/A	N/A	KR903621^8^	N/A	KR903269^8^	KR904147^8^
*Gryllus bimaculatus* De Geer, 1773	x	x	AF248685^2^	AY905292^5^	AF248659^2^	N/A	KU705555*	KR903504*	N/A	KR903154*	AF514509^6^
*Homeogryllus orientalis* Desutter, 1985		x	KR903752^8^	KR903925^8^	KR903413^8^	N/A	N/A	KR903586^8^	N/A	KR903235^8^	KR904112^8^
*Lebinthus lifouensis* Desutter-Grandcolas, 1997	x	x	AY905309^5^	AY905279^5^	AY905364^5^	KU705566*	KU705556*	KU705583*	N/A	KU705599*	AY905336^5^
*Lebinthus luae* Robillard & Tan, 2013	x	x	JF972524^4^	KR904017*	JF972493^4^	KU705567*	KU705557*	KR903665*	KU705615*	KR903321*	KR904199*
*Lebinthus nattawa* Robillard, 2009	x	x	JF972525^4^	JF972510^4^	JF972494^4^	KU705568*	KU705558*	KU705584*	N/A	KU705600*	JF972541^4^
*Lebinthus santoensis* Robillard, 2009 P	x		KU705528*	KU708011*	KU5535*	KU705569*	N/A*	KU705585*	N/A	KU705601*	KU705543*
*Lebinthus santoensis* Robillard, 2009 V	x	x	JF972527^4^	JF972511^4^	JF972495^4^	JX897405^1^	JX897441^1^	JX897467^1^	N/A	JX897548^1^	JF972542^4^
*Lebinthus* sp. PNG	x	x	JF972528^4^	JF972513^4^	KU705536*	KU715289*	KU715288*	KU715290*	KU715292*	KU715291*	JF972544^4^
*Lebinthus villemantae* Robillard, 2010	x	x	JF972526^4^	JF972512^4^	JF972496^4^	KU705570*	KU705559*	KU705586*	N/A	KU705602*	JF972543^4^
*Luzaridella obscura* Desutter-Grandcolas, 1992		x	KR903708^8^	KR903871^8^	KR903365^8^	N/A	N/A	KR903536^8^	N/A	KR903186^8^	KR904061^8^
*Microlandreva* sp. Chopard, 1958		x	KR903782^8^	KR903960^8^	KR903442^8^	N/A	N/A	KR903620^8^	N/A	KR903268^8^	KR904146^8^
*Nisitrus vittatus* (Haan, 1842) Se	x		AY905314^5^	AY905284	AY905369^5^	KU705571*	N/A	KU705587*	N/A	KU705603*	AY905340^5^
*Nisitrus vittatus* (Haan, 1842) Si	x	x	AY905314^5^	AY905284	AY905369^5^	KU705572*	N/A	KR903667*	JN887883^7^	JX897546^1^	KR904201^5^
*Ornebius xanthopterus* Guérin-Méneville, 1844		x	KR903769^8^	KR903944^8^	KR903430^8^	N/A	N/A	KR903605^8^	N/A	KR903254^8^	KR904131^8^
*Paranisitra longipes* Chopard, 1925	x	x	KR903827*	KR904020*	N/A	KU715287*	N/A	KR903668*	N/A	KR903325*	KR904202*
*Pixibinthus sonicus* Anso & Robillard, sp. nov. FN1	x		KU705531*	KU708014*	KU705539*	KU705573*	N/A	KU705589*	N/A	KU705604*	N/A*
*Pixibinthus sonicus* Anso & Robillard, sp. nov. FN2	x		KU705532*	KU708015*	KU705540*	KU705574*	N/A	KU705590*	N/A	KU705605*	KU705547*
*Pixibinthus sonicus* Anso & Robillard, sp. nov. GK1	x	x	KU705529*	KU708017*	KU705537*	KU705575*	N/A	KU705591*	N/A	KU705606*	KU705545*
*Pixibinthus sonicus* Anso & Robillard, sp. nov. GK2	x		KU705534*	KU708016*	KU705542*	KU705576*	N/A	KU705593*	N/A	KU705607*	KU705549*
*Pixibinthus sonicus* Anso & Robillard, sp. nov. RB1	x		KU705530*	KU708012*	KU705538*	KU705577*	N/A	KU705592*	N/A	KU705608*	KU705546*
*Pixibinthus sonicus* Anso & Robillard, sp. nov. RB2	x		KU705533*	KU708013*	KU705541*	KU705578*	N/A	KU705594*	N/A	KU705609*	KU705548*
*Pteroplistes masinagudi* Jaiswara, 2014		x	KR903693^8^	KR903854^8^	KR903349^8^	N/A	N/A	KR903521^8^	N/A	KR903170^8^	KR904045^8^
*Swezwilderia* sp.	x	x	JF972529^4^	JF972514^4^	JF972498^4^	KU705579*	N/A	N/A	N/A	KR903327*	JF972545^4^
*Xenogryllus marmoratus* Bolívar, 1890	x	x	KR903830*	KR904024*	KR903491*	N/A*	KU705560*	N/A	KU705610*	KR903329*	KR904206*

The molecular sampling of dataset 2 was composed of a sub-sampling of dataset 1 to reduce each ingroup species to a single individual, except *Cardiodactylus novaeguineae* that occurs in New Caledonia and other Pacific islands (Tables 1 and 2). We also increased the species sampling of the Grylloidea superfamily in order both to better estimate divergence times and to use an appropriate calibration point (see below). We consequently added representatives of the major Gryllidea lineages delimited in Chintauan-Marquier et al. [37]:Trigonidiidae (two species), Pteroplistinae (one species), Phalangopsidae (four species), and Gryllidae (six species). We finally used as most external outgroups, one species belonging to Mogoplistidae (Grylloidea) and one species of Gryllotalpidae (Gryllotalpoidea; Tables 1 and 2).

#### Selection of DNA regions

We selected nine DNA loci, five mitochondrial and four nuclear, proved to have been useful in previous phylogenetic studies on Grylloidea [16,27,37,40,41]. These are fragments of the small (12S rRNA, ~ 400 bp) and large (16S rRNA, ~ 500 bp) mitochondrial ribosomal subunits, the mitochondrial gene coding for cytochrome b protein (cytb, ~ 400 bp), fragments of the mitochondrial cytochrome oxidase subunits 1 (CO1, ~ 750 bp) and 2 (CO2, ~ 400 bp) genes, a fragment of the small nuclear ribosomal subunit (18S rRNA, ~ 650 bp), a fragment of the large nuclear ribosomal subunit (28S rRNA, ~ 400 bp), and fragments of the genes coding for H3 protein (H3, ~ 330 bp) and elongation factor 1-α (EF1α, ~ 950 bp). We used these nine DNA loci for analyses of dataset 1, to refine and improve the internal phylogenetic resolution. However we observeda high level of missing sequences for the CO1 and CO2 regions, especially for outgroup species of dataset 2. We therefore used only the seven following loci for phylogenetic analyses of this dataset: 12S, 16S, 18S, 28SA, cytb, EF1a, and H3.

#### Laboratory procedures and Preparation of datasets

Molecular work was performed at the Service de Systématique Moléculaire of the MNHN. All sequences were generated by using the DNA extraction, amplification and sequencing protocols of the nine DNA loci, described in Nattier at al. [27]. Some DNA extractions were made from median legs of crickets using the Epmotion 5075 robot (Eppendorf). In total we generated 106 new DNA sequences (33% of the total samplings) for this study (Tables 1 and 2), while other sequences were generated in previous studies and downloaded from Genbank [27,37,38,39,40,41,42,43,44]. Primers and annealing temperatures for each DNA locus are given in S1A Table. Newly generated sequences were edited in Sequencher v.4.9 (Gene Codes Co.) and BioEdit v.7.0.5.3 [45], blasted with NCBI blast tools, and submitted to GenBank (Tables 1 and 2).

Multiple alignments were generated for each DNA locus using the software Muscle v.3.7 [46] with default parameter settings, by using the online portal Phylogeny.fr [47]. Subsequently we adjusted them manually. We concatenated the different DNA locus partitions with SequenceMatrix v.1.7.7 [48]. The datasets 1 and 2 resulted in a combined alignment of ~4750 bp and 3789 bp, respectively (S1B and S1C Table). Several ambiguous and large indel regions in the alignments of loci 12S, 16S, 18S, and EF1α were found within the dataset 2, and attributed to the inclusion of several distantly related Grylloidea taxa. This induced problematic homology hypotheses, and we consequently removed them from the DNA alignments (S1 Table).

#### Phylogenetic analyses

Preliminary single-locus and combined phylogenetic inferences were carried out using maximum likelihood (ML) and Bayesian Markov Chain Monte Carlo (MCMC) analyses for both datasets 1 and 2. Best-fit model for each DNA locus was identified using jModelTest v.2.1.3 [49] based on the Akaike Information criterion (S1B and S1C Table). The combined datasets were partitioned to allow each locus to have its specific model parameters [50,51].

The ML analyses were performed in RAxML v.7.4.2. [52,53] by running 500 likelihood searches. To evaluate node support, a supplementary bootstrap analysis (BS; [54]) was performed using 500 replicates. A clade with a BS value > 95% was considered as well supported.

The Bayesian MCMC analyses were performed using MrBayes v.3.2.6 [51]. The single-locus and combined analyses were set as follows: four Metropolis-coupled Markov chains with an incremental heating temperature of 0.2 were run for 50 and 100 million generations with a tree sampled every 10000th generation for dataset 1 and dataset 2, respectively. The analysis was repeated four times for both datasets, all starting with random trees. Bayesian MCMC analyses were carried out using the CIPRES Science Gateway v.3.3 [55]. The MCMC sampling was considered sufficientwhen the effective sampling size (ESS) was higher than 200, as verified in Tracer v.1.6 [56], or when the potential scale reduction factor was reasonably close to 1.0 for each parameter (PSRF; [57]). After a burn-in period of 5× 10^6^ and 10 × 10^6^ for dataset 1 and dataset 2, respectively, the remaining trees were used to construct a majority rule consensus tree and its associated Bayesian posterior probabilities (PP). A clade with a PP value higher than 0.95 was considered as well supported. Trees were finally visualized with FigTree v.1.4.0. (http://tree.bio.ed.ac.uk/software/figtree/).

#### Bayesian divergence times estimates

The temporal evolution of *Pixibinthus* and its close relatives was estimated using the Bayesian MCMC approach implemented in BEAST v.1.8.0 for dataset 2 [58]. DNA loci were combined and partitions were set as in the preliminary Bayesian MCMC analyses (see above). We unlinked all DNA site models to analyze each locus under separate substitution models. An uncorrelated relaxed molecular clock model was selected to allow estimates of independent rate variation across branches following a lognormal distribution. The Birth-Death speciation process was implemented for the tree prior, assuming constant speciation and extinction rates per lineage. The mean of the branch rates (UCLD.mean) was set to follow a lognormal distribution with a log (mean) set to -4 and a log (standard deviation) set to 1. We started our analysis with a random tree. The MCMC was run for 100 million generations and sampled every 10000th generations. The analysis for each dataset was conducted three times, and was carried out using the CIPRES Science Gateway v.3.3 [55]. Convergence of runs and adequate MCMC sampling were checked using Tracer v.1.6 [56]. The first 10 × 10^6^ generations of each run were manually discarded as burn-in. The remaining trees were summarized using a Maximum Clade Credibility target tree in TreeAnnotator v.1.8.0 [58], as well as Bayesian posterior probability (PP), median height (= age estimate) and the 95% highest posterior density heights interval (95% HPD) of each node.

#### Calibration point

Divergence dates were previously estimated to test biogeographical hypotheses in Eneopterinae crickets by Nattier et al. [40]. Age estimates were mainly obtained with several geological calibration points, considering that few fossils or dated trees were available at that time. Since that study, Song et al. [59] produced a large dated tree for Orthoptera which provided a secondary calibration for the present study in order to avoid the generally less accurate geological calibrations. We choose to use the secondary calibration point which corresponds to the divergence time between the Grylloidea and Gryllotalpoidea superfamilies. This calibration was assigned to the root node of our majority rule consensus tree generated in the preliminary Bayesian MCMC analyses of dataset 2. It corresponds to the divergence time between our most external outgroup species, *Gryllotalpa* sp. and all the other Grylloidea species of our dataset 2 sampling. This calibration was set to follow a uniform distribution, and by using a lower bound of 220 Myr and an upper bound of 250 Myr, which correspond to the 95% HPD of this divergence time, with a median value of 233.99 Myr [59].

### Species richness and relative diversification rates

We tested the difference in species richness between *Pixibinthus* and its sister-clade (see below) using Equation (3) of Slowinski & Guyer [60]. To allow calculating this estimate and to ensure that the first basal split of this sister-clade in dating tree was assessed, we preliminary estimated its species richness, according to Desutter-Grandcolas & Robillard [26], Robillard et al. [61], and our own knowledge.

### Ensemble species distribution modelling

Ensemble Species Distribution Model (ESDM) analyses were carried out using combined R packages, and based on nine algorithms (GLM, GAM, MARS, GBM, CTA, RF, MAXENT, ANN and SVM) in order to test if the contemporary geographical range of *Pixibinthus* could have been more expanded, and would have been constrained by environmental changes. Dataset was composed of the coordinates of field sampling sites where *Pixibinthussonicus* has been recorded. Nine GIS ecological layers were used for the modelling analyses. Uncorrelated variables were selected among the 19 bioclimatic variables available in New Caledonia from worldclim database [62]leading to the selection of seven bioclimatic variables (BIO1, 2, 3, 4, 7, 12 and 15) at 30arc-second [62]. We also included altitude from Digital Elevation Model at 10m resolution, and main vegetation units distribution map available at 300m resolution. All layers were rescaled with a 300 m resolution. We used 30% randomly selected occurrence data as a testing dataset. The consensus model was built by summing algorithms suitability maps weighted with their area under the receiving operating characteristic (ROC) curve (AUC), only for model with an AUC > 0.75 [63]. To evaluate the relative contribution of each variable to the model Pearson's correlation coefficient between the full model and one without each variable is measured [64].

## Results

### Systematics and taxon description

The new genus and the new species are described here. Habitat, life history traits and calling song are also precisely defined.

**Order**: Orthoptera

**Family**: Gryllidae

**Subfamily**: Eneopterinae

**Tribe**: Lebinthini

**Genus**:***Pixibinthus* Robillard and Anso**, **gen**. **nov**.

urn:lsid:zoobank.org:act:94405C62-C382-407F-BAC0-4B631EC5A0D3

**Type species**: *Pixibinthussonicus* Anso & Robillard, sp. nov.

**Etymology**: Genus named after ‘Pixie’, the diminutive mythical creatures of folklore, hidden by nature, close but unseen, as these crickets which were never encountered by previous authors despite past extensive studies on crickets in New Caledonia.

**Diagnosis**: Among Eneopterinae and Lebinthini genera, *Pixibinthus* is characterized by a diminutive size, smaller than all previously described species of *Lebinthus* from the Loyalties and Vanuatu (*L*. *lifouensis* Desutter-Grandcolas, 1997 and *L*. *santoensis* Robillard, 2009 respectively). For numerous aspects, the new genus appears as an intermediate form between *Lebinthus* and *Agnotecous*. It resembles these genera by microptery in both sexes (FW short and hind wings absent), but differs by the following characters: head shape more rounded with smaller eyes and wider fastigium; in dorsal view, combined width represents 35% of head width, against 50% in *L*. *santoensis* and 46% in *L*. *bitaeniatus*; head rounded in facial view, contrary to the triangular shape in *Lebinthus*; ocelli very small compared to *Lebinthus*; male FWs with dorsal field and lateral field of similar length, unlike in *Agnotecous*, but as in *Lebinthus*; female FWs as long as in males, unlike in most *Lebinthus* species, slightly overlapping.

**Fig 1:**
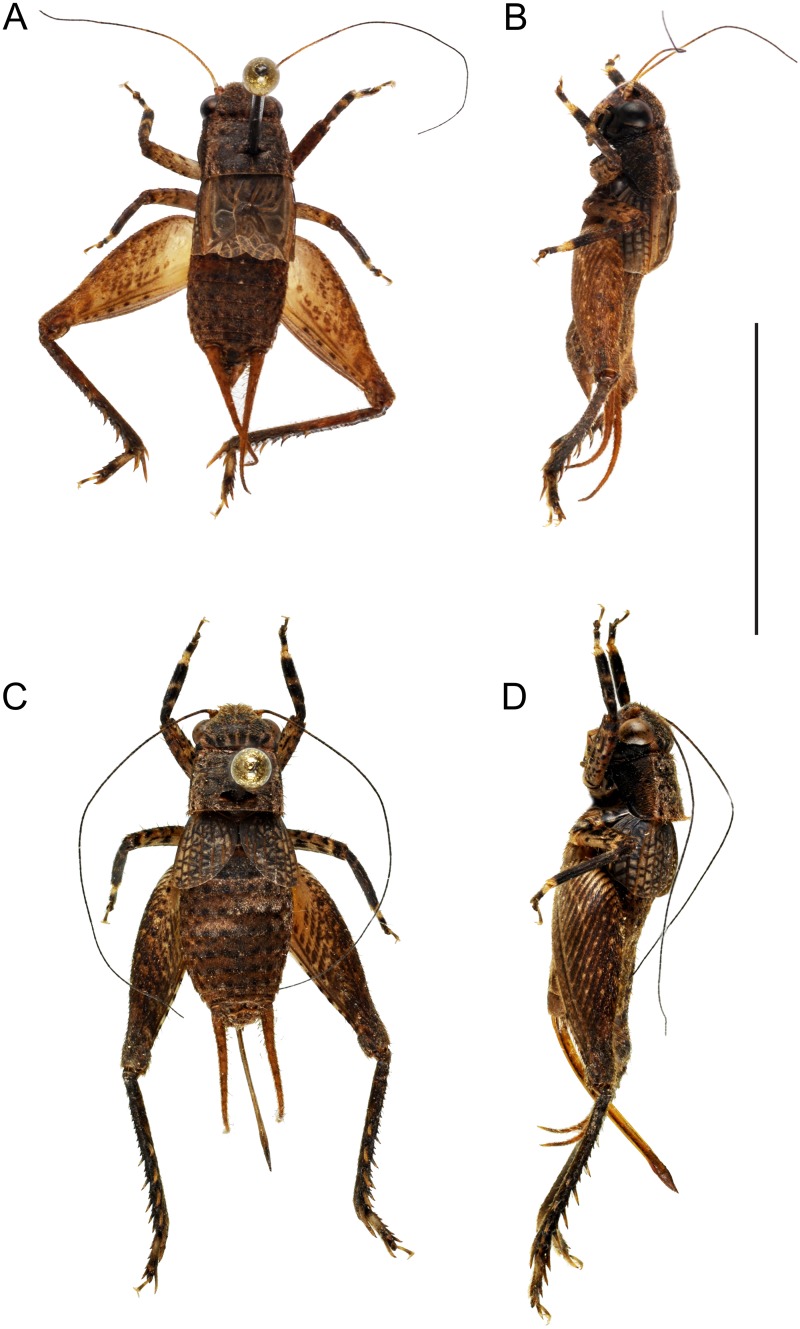
Dorsal and lateral views of *Pixibinthus sonicu*s. (A) & (B) Male. (C) & (D) Female. Scale bar = 5 mm.

**Description**: Genus characterized by its small size. Head shape uncommon, fastigium wider than long, three times as wide as scape. Eyes small, little prominent; Ocelli very small. Pronotum dorsal disk almost rectangular, wider than long, its posterior margin straight. Legs rather short. Fore tibiae with two tympana; inner tympanum covered by a sclerotized expansion (Fig 1A), its membrane visible along a small longitudinal slit only; outer tympanum ellipsoidal, transversally plicate. Fore tibiae with two inner and one outer apical spurs. Tibiae II with two inner and two outer spurs. Hind femora very wide and muscular. Hind tibiae serrulated on their whole length, not furrowed longitudinally, with four pairs of subapical spurs and three pairs of apical spurs; inner spurs long and curved, outer spurs short and straight. Tarsomeres III-1 with two dorso-apical spines and a row of spines on outer dorsal edge; one lateral outer spine.FWs short, not reaching abdomen mid-length; hindwings absent.

**Fig 2:**
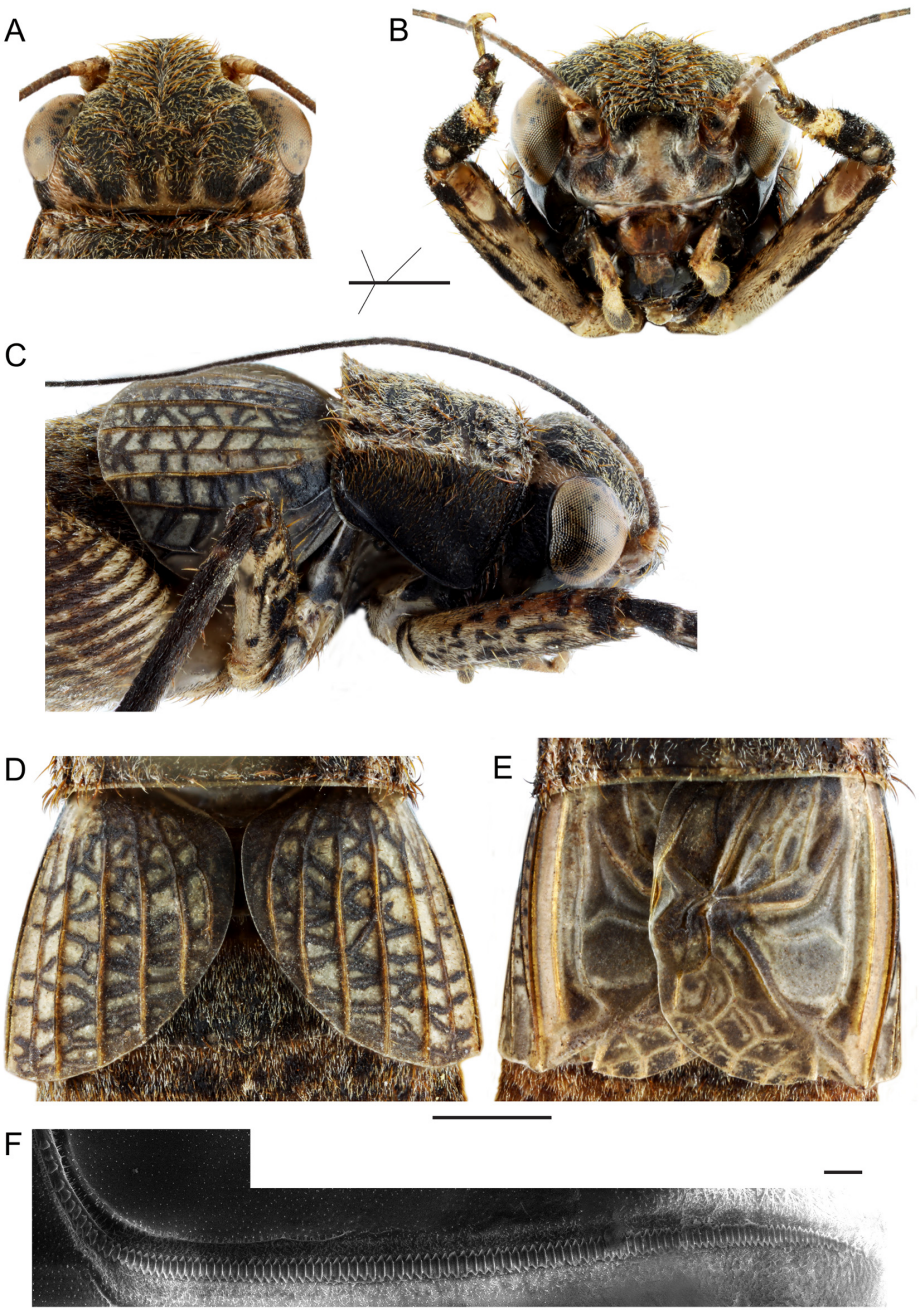
Detailed morphology of *Pixibinthus sonicus*. (A)Dorsal view of the female face. (B) Facial of the female face. (C) Lateral view of the anterior body part of female. (D) Dorsal view of detailed FWs for female. (E) Dorsal view of detailed FWs for male (E). (F) Stridulatory file with SEM. Scale bars = 1 mm for (A), (B), (C), (D), (E); 50 μm for (F).

**Fig 3:**
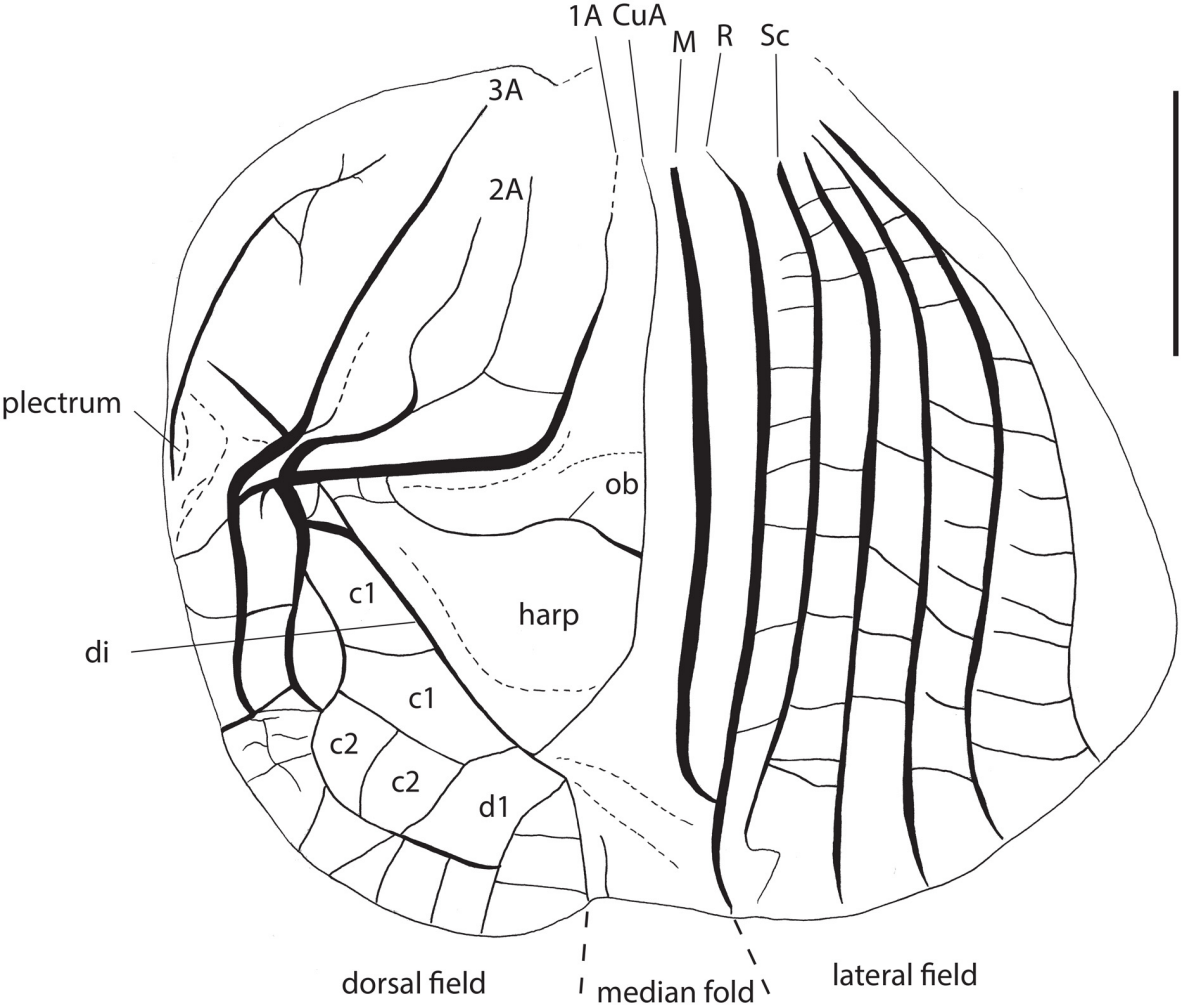
Male forewing venation of *Pixibinthus sonicus*. For abbreviations and symbols, see Material and methods. Scale bar = 1 mm.

**Male**: Metanotal glands absent. Lateral and dorsal fields of FWs of similar lengths (Figs 2 and 3). 1A vein (file) with a marked angle (only curved in *Lebinthus*). Harp with one bisinuated oblique vein. Mirror and d2 cell not differentiated from other cells of D alignment. Stridulatory file with teeth both on transverse and longitudinal parts of 1A. Diagonal vein well visible. CuP absent but claval fold visible. CuA faint, slightly curved inward. Median fold small, located on dorsum as in *Lebinthus* and *Agnotecous*. Chord cells differentiated. Apical field including only few cells posterior to mirror (alignment E). Subgenital plate clog-shaped, twice as long as sternites; inner side of subgenital plate with lateral swellings.

**Fig 4:**
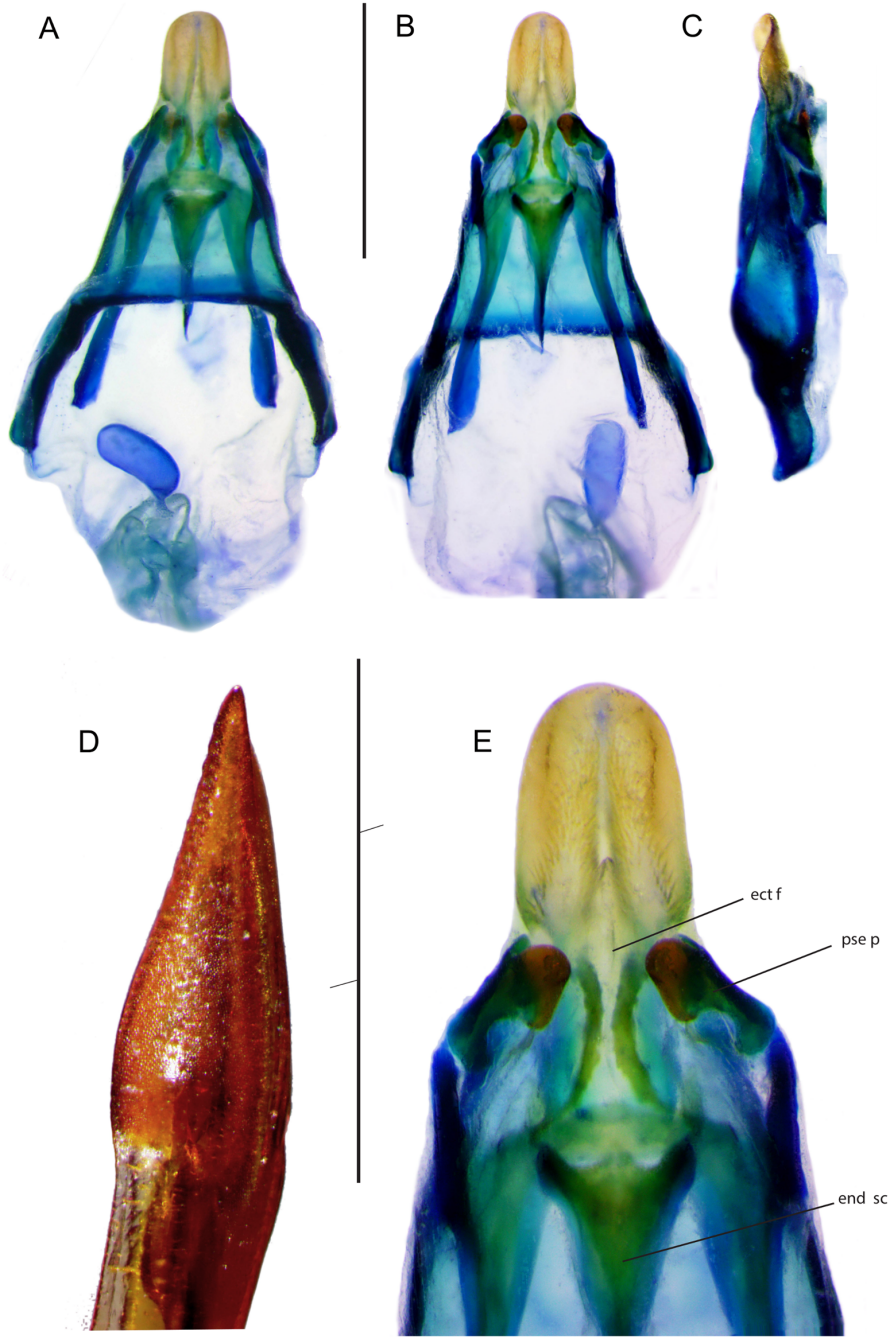
Genitalia of *Pixibinthus sonicus*. (A) Dorsal, (B) ventral, and (C) lateral views of male genitalia. (D) Lateral view of apex of female ovipositor. (E) Ventral view of male genitalia. For abbreviations and symbols, see Material and methods. Scale bars = 1 mm.

**Male genitalia**: Pseudepiphallus triangular, basal margin straight, posterior apex elongate, without paired lophi and forming a wide gutter (Fig 4). Rami straight, parallel and short. Ventral pseudepiphallic plate wide. Pseudepiphallic parameres sclerotized, convergent, their basis strong, with two posterior lobes, one oriented dorsally and one forming a rounded ventral plate, and a basal membranous lobe. Ectophallic apodemes parallel and long, their apex lamellate. Ectophallic arc well sclerotized, wide and slightly curved posteriorly, with a small medio-posterior expansion. Ectophallic fold long, with elongate lateral sclerites forming a “)(“pattern; its apex triangular and membranous. Endophallic sclerite large, comprising posteriorly a short median expansion and lateral arms; sclerite very long anteriorly, exceeding pseudepiphallic sclerite. Endophallic apodeme with well-developed lateral lamellas and a narrow dorsal crest.

**Female**: FWs as long as in males (Fig 2), slightly overlapping, dark brown with whitish veins; dorsal field with 7 strong longitudinal veins; lateral field with 4 longitudinal veins; Sc without bifurcations. Ovipositor rather short, as long as hind femur, laterally flattened, apex lanceolate and slightly denticulate dorsally (Fig 4D).

**Female genitalia**: Copulatory papilla little sclerotized, with a basal sclerite forming a wide ring; apex little differentiated, slightly indented andfolded ventrally.

***Pixibinthus sonicus* Anso and Robillard**, **sp**. **nov**.

urn:lsid:zoobank.org:act:CA5D7BDC-7504-435C-8B67-5BDCF11987BB

**Type material: Holotype male**: New Caledonia, Grande Terre, Forêt Nord, milieu paraforestier, 22.191554 S 166.5608 E, 100 m, 1.XII.2013-12.II.2014, PAFN1-24, jour, litière, J. Anso (MNHN-EO-ENSIF244). **Allotype female**: Same locality, date and collector as HT, PAFN2-4, jour, litière (MNHN-EO-ENSIF71). **Paratypes**: New Caledonia, Grande Terre, Pic du Grand Kaori, 22.16471 S 166.53399 E, 260 m, 11.II.2014, 1♂, AGN1, jour, litière, J. Anso (MNHN-EO-ENSIF98); XI.2013, 1♀, Lfem1, J. Anso (MNHN-EO-ENSIF124). Forêt Nord, milieu paraforestier, 22.191554 S 166.5608 E, 100 m, 1.XII.2013-12.II.2014, J. Anso: 1♂, PAFN1-46, jour, litière (MNHN-EO-ENSIF70); 1♀, PAFN2-23, (MNHN-EO-ENSIF90); 1♀, PAFN2-2, jour, litière (IAC); 1♀, PAFN2-3, jour, litière (MNHN-EO-ENSIF144); 1♂, PAFN2-12, nuit, litière (IAC); 1♂, PAFN2-24, jour, litière (Nouméa); 1♀, PAFN2-33, nuit, litière (MNHN-EO-ENSIF361); 1♀, PAFN1-37, nuit, litière (MNHN-EO-ENSIF142); 1♂, PAFN1-38, nuit, litière (MNHN-EO-ENSIF81); 1♀, PAFN2-52, nuit, litière (MNHN-EO-ENSIF123); 1♀, PAFN1-1, jour, litière (MNHN-EO-ENSIF77); 1♂, PAFN1-13, jour, litière, molecular sample L66LeNCFN2 (MNHN-EO-ENSIF83); 1♀, PAFN1-15, jour, litière (Nouméa); 1♂, PAFN1-23, jour, litière, molecular sample L60LeNCFN1 (MNHN-EO-ENSIF99); 1♀, PAFN1-22, jour, litière (MNHN-EO-ENSIF127); 1♀, PAFN1-40, jour, litière(MNHN-EO-ENSIF362); 1♀, PAFN1-42, jour, litière (MNHN-EO-ENSIF118); 1♂, PAFN1-51, nuit, litière (MNHN-EO-ENSIF128). Nouvelle-Calédonie, Grande Terre, Pic du Grand Kaori, 22.16471 S 166.53399 E, 260 m, maquis, VI.2013, adultes en élevage, J. Anso: 3 males (MNHN-EO-ENSIF149, 150, 153); 4 males, enregistrement appel F0-males1-4 (MNHN-EO-ENSIF158, 154, 152, 125), 4 females, (MNHN-EO-ENSIF129, 132, 139, 140).

**Additional material examined**: Parc de la Rivière Bleue, maquis, 22.08375 S 166.424776 E, 200 m, 11-19.III.2014: 1 male, MARIV1-1, nuit, litière, molecular sample L59LeNCRBa1 (MNHN-EO-ENSIF105); 1 juvenile, MARIV1-15, jour, litière, molecular sample L67LeNCRBa2 (MNHN-EO-ENSIF133); 7 juveniles, MARIV1-3, 6, 7, 12, 13, 14, 19, litière (MNHN); 5 juveniles, MARIV2-2, 3, 7, 8, 9, litière (MNHN). Forêt Nord, milieu paraforestier, 22.191554 S 166.5608 E, 100 m, 1.XII.2013-26.II.2014: 12 juveniles, PAFN2-9,14,25,48,51,53,56, PAFN1-47,52,58,59,60, nuit, litière; 3 juveniles, PAFN2-49, PAFN1-16,45, jour, litière; 1♀, PAFN1-14, jour, litière; 1♀, PAFN1-7, nuit, litière (MNHN). Chute de la Madeleine, maquis, 22.23568 S 166.85268 E, 255m, 10-26.II.2014, J. Anso: 1 juvenile, MAMAD1-24, molecular sample L116PixMad1 (MNHN-EO-ENSIF65); 4 juveniles, MAMAD1-11, 24, 26, litière (MNHN); 3 juveniles, MAMAD2-7, 9, 22, litière (MNHN).

**Type locality**: New Caledonia, Grande Terre, Forêt Nord.

**Etymology**: Named after the very high dominant frequency of the species’ calling song.

**Fig 5:**
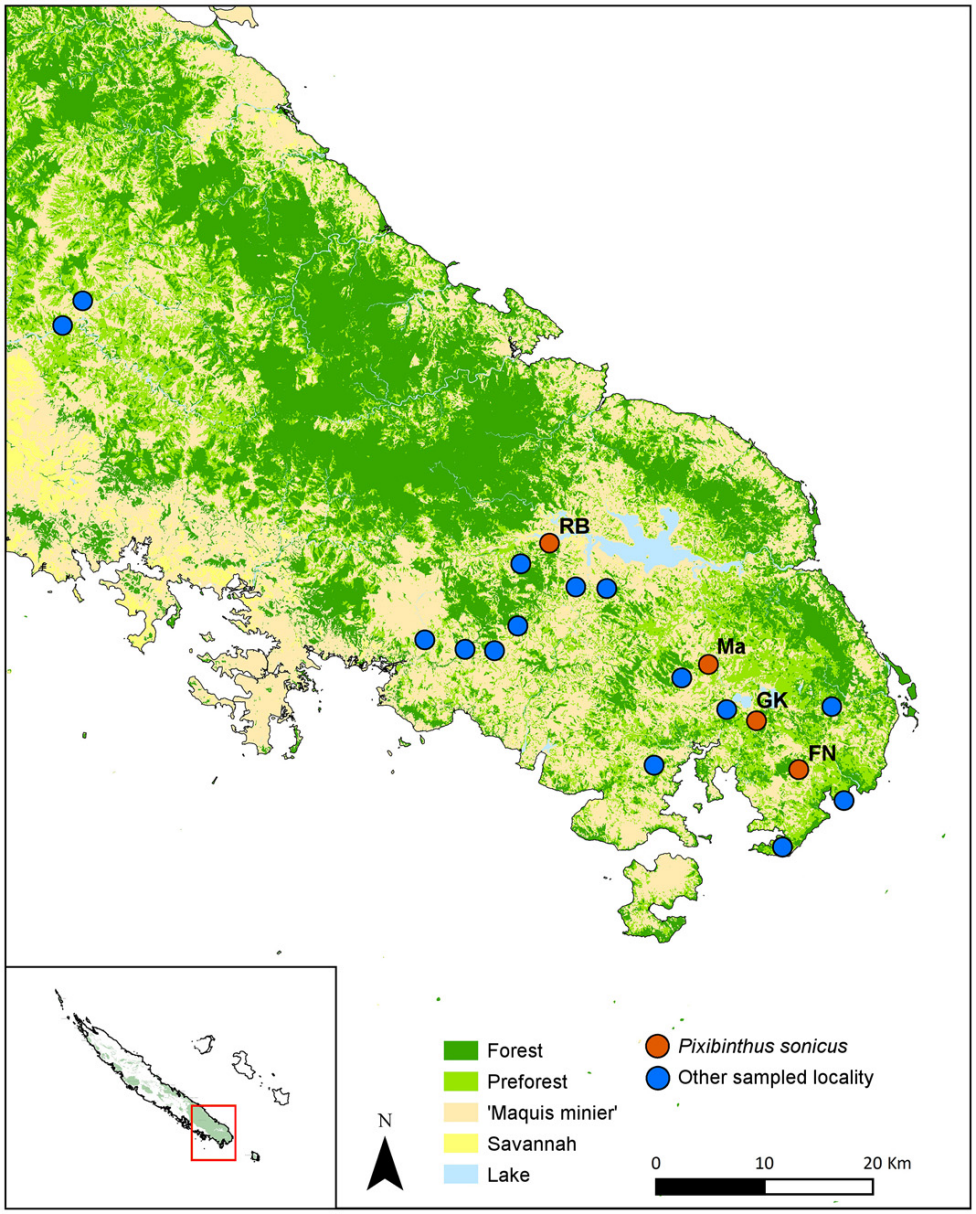
Map of sampled localities in the southern part of New Caledonian Grande Terre. Blue dots indicate localities where populations of *Pixibinthus sonicus* were not found. Orange dots indicate localities where *P*. *sonicus* was found.

**Distribution**: New Caledonia, Grande Terre, Province Sud: Rivière Blanche, Chute de la Madeleine, Pic du Grand Kaori, Forêt Nord (Fig 5).

#### Diagnosis

*Pixibinthus sonicus* is recognized by its small size, face light brown with seven black spots, male genitalia, and high dominant frequency of calling song.

**Description**: In addition to the characters of the genus: Size very small, stocky shape. Coloration: No contrasted sexual dimorphism (Fig 1A & 1C). Body mostly brown to dark brown, slightly contrasted with lighter parts on legs. Head dorsum with six wide dark brown bands visible basally and fused between the eyes; fastigium dark brown; area posterior to eyes yellow; cheeks entirely shiny black;face light brown, with seven characteristic black spots: four forming a square on the front head, one above epistomal suture, and two bellow antennal pits; spots variable in size and connectivity, sometimes more or less fused together in a complex pattern. Maxillary palpi: article 1–3 black, articles 4–5 lighter with apical darker ring on article 5. Pronotum brown to dark brown, with lighter lateral margins with small black dots; lateral lobes of pronotum homogeneously black. Pronotum dorsal disk and lateral lobes separated by a short concave yellow line. Legs: Tibiae I black with three small lighter flecks; tibiae II entirely black with a whitish apical ring; tibiae III dark brown; femora III yellowish with dark brown diagonal stripes strongly contrasted on external face; femora I and II yellowish with small brown flecks; first and third tarsomeres I-III dark yellowish basally, their apex dark brown. Abdomen brown, darker in apical part, with one yellowish small dot for each segment. Cerci yellowish, with small scattered darker flecks.

#### Male

FW mostly gray to dark brown with a pale area including bases of CuA, M and R veins. Area between M and R whitish, veins orange brown. FW venation: 1A with 110 stridulatory teeth, 12 on the basal angle and 98 on the transverse part of the file (n = 1; Fig 2F). Cell c1 with a faint median transverse vein; c2 rectangular. M yellow brown, curved posteriorly, fused to R near FW apex, very thin near fusion; R straight, strong along its whole length. FW lateral field including Sc and 3–4 strong longitudinal veins. Hind tibia inner serrulation (n = 3): no spine between the apical and the first subapical spur; one spine between subapical spurs 1 and 2; two spines (1–2) between spurs 2 and 3; two spines between subapical spurs 3 and 4; seven spines (4–10) above subapical spur 4; outer serrulation: no spine between apical and subapical spurs; one spine between subapical spurs 1 and 2; two spines (1–3) between subapical spurs 2 and 3; two spines (2–3) between subapical spurs 3 and 4; seven spines (6–8) above subapical spur 4. Tarsomeres III-1: four outer (3–4) and no inner spines, in addition to apical spines.

#### Female

Hind tibia inner serrulation (n = 3): no spine between the apical and the first subapical spur; one spine between subapical spurs 1 and 2; two spines (1–2) between spurs 2 and 3; one spine (1–2) between subapical spurs 3 and 4; four (4–5) spines (4–10) above subapical spur 4; outer serrulation: no spine between apical and subapical spurs; one spine between subapical spurs 1 and 2; two spines between subapical spurs 2 and 3; two spines between subapical spurs 3 and 4; eight spines above subapical spur 4. Tarsomeres III-1: three outer and no inner spines, in addition to apical spines.

#### Measurements

See Table 3.

**Table 3:** Measurements of *Pixibinthus sonicus* sp. nov.(in mm).

	PronL	PronW	FWL	FWW	FIIIL	FIIIW	TIIIL	TIII spines				TaIIIs	OL
								Ias	Ibs	Oas	Obs		
Male holotype	2	2.3	3.3	2.6	6.8	2.4	5.3	5	6	8	5		
Males paratype (n = 5)	1.8–2.1	2.4–2.7	2.8–3.5	1.8–2.0	6.1–7.1	2.1–2.5	4.5–5.5	4–6	3–6	8–11	5–6	3–6	
(Male mean)	(2.0)	(2.5)	(3.1)	(2.0)	(6.7)	(2.3)	(5.2)	(5)	(4)	(9)	(5)	(3)	
Allotype female	1.7	2.5	2.1	-	6.5	2.4	5.3	3	4	8	5		5.5
Females paratype (n = 5)	1.7–2.1	2.1–2.8	2.0–2.4	-	6.5–8.1	2.2–2.7	5.1–5.6	4–6	3–5	7–9	4–6	3–4	5.5–7.0
(Female mean)	(1.9)	(2.5)	(2.2)	-	(7.0)	(2.4)	(5.4)	(5)	(4)	(8)	(5)	(4)	(6.4)

#### Juvenile

Body color light brown mottled with dark brown to black spots. Head, including face, pronotum and legs with similar color patterns as in adults.

**Fig 6:**
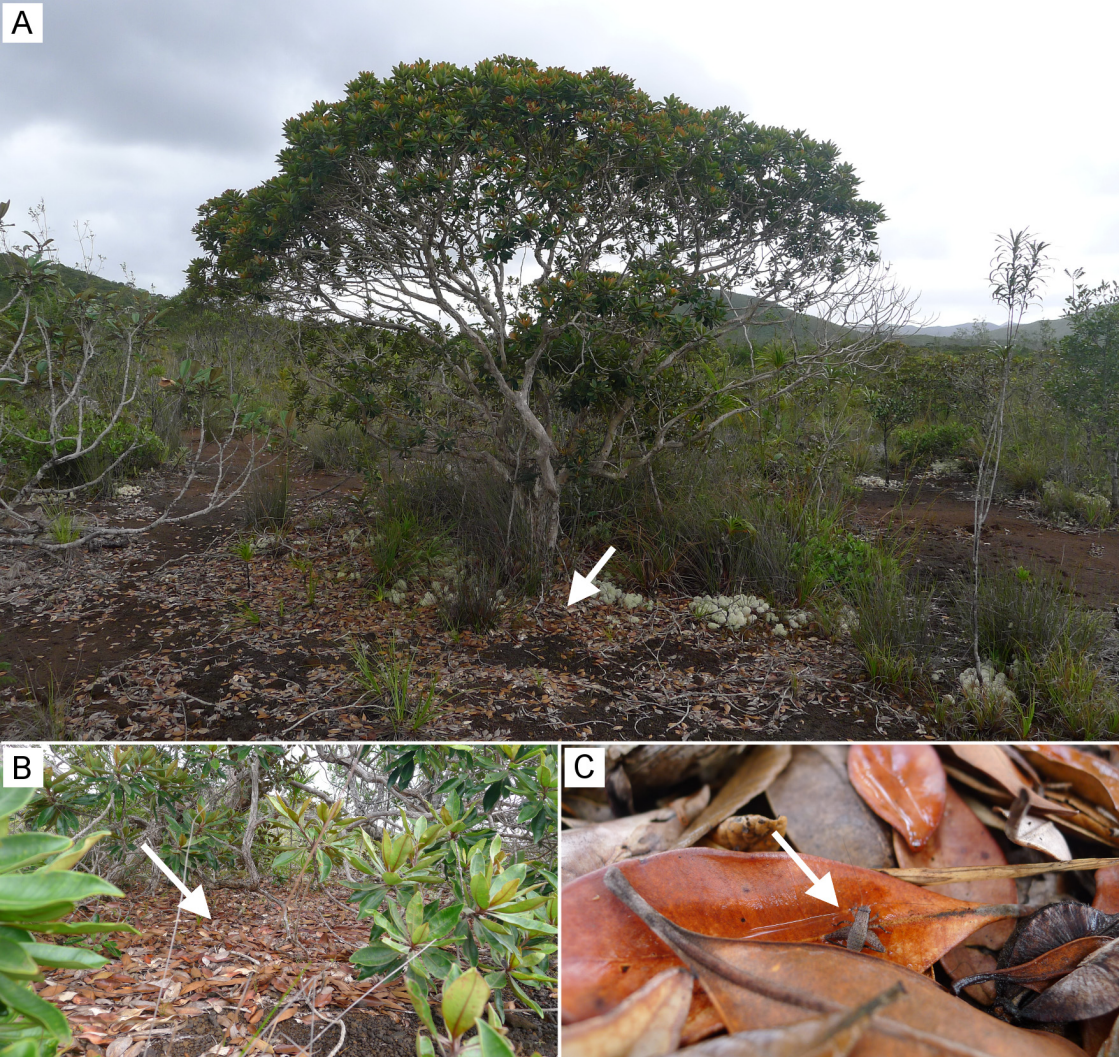
Habitat of *Pixibinthus sonicus*. (A)Low sclerophyllous ‘maquis minier’ around les Chutes de la Madeleine. (B) Detailed view of habitat in leaf litter. (C) Juvenile of *P*. *sonicus* (size: approximately 1cm).

**Habitat and life history traits**: *Pixibinthus sonicus* was clearly identified as a new monotypic genus and new species after intensive sampling on various habitats, in the southern part of New Caledonia, representing an area of about 2150 km^2^. Among 19 sampled localities, including shrubby vegetation, preforest and rainforest, *P*. *sonicus* was only found in low to tall sclerophyllous shrubland and was clearly absent from forested areas (Fig 6). Four populations of *P*. *sonicus* were identified in our study sites. Three populations were found in low sclerophyllous ‘maquis minier’, with dominant strata lower than 5–6 meters. One population was found in tall sclerophyllous ‘maquis miniers’ at Forêt Nord (22.32259 S 166.93134 E), 6 km away from the population of Pic du Grand Kaori, separated by high ridges. Adults and juveniles were both collected during day and night, in the leaf litter. In these three localities, abundances were relatively small with a dozen of specimens collected in each site.

**Behavior**: Males of *Pixibinthus sonicus* emit their calling song starting from 16:30 PM until next morning at 03:00 AM, as attested by 72 hours ambient recording with SM2 Bat; this activity would characterize *P*. *sonicus* as a nocturnal species.

### Acoustic description

**Fig 7:**
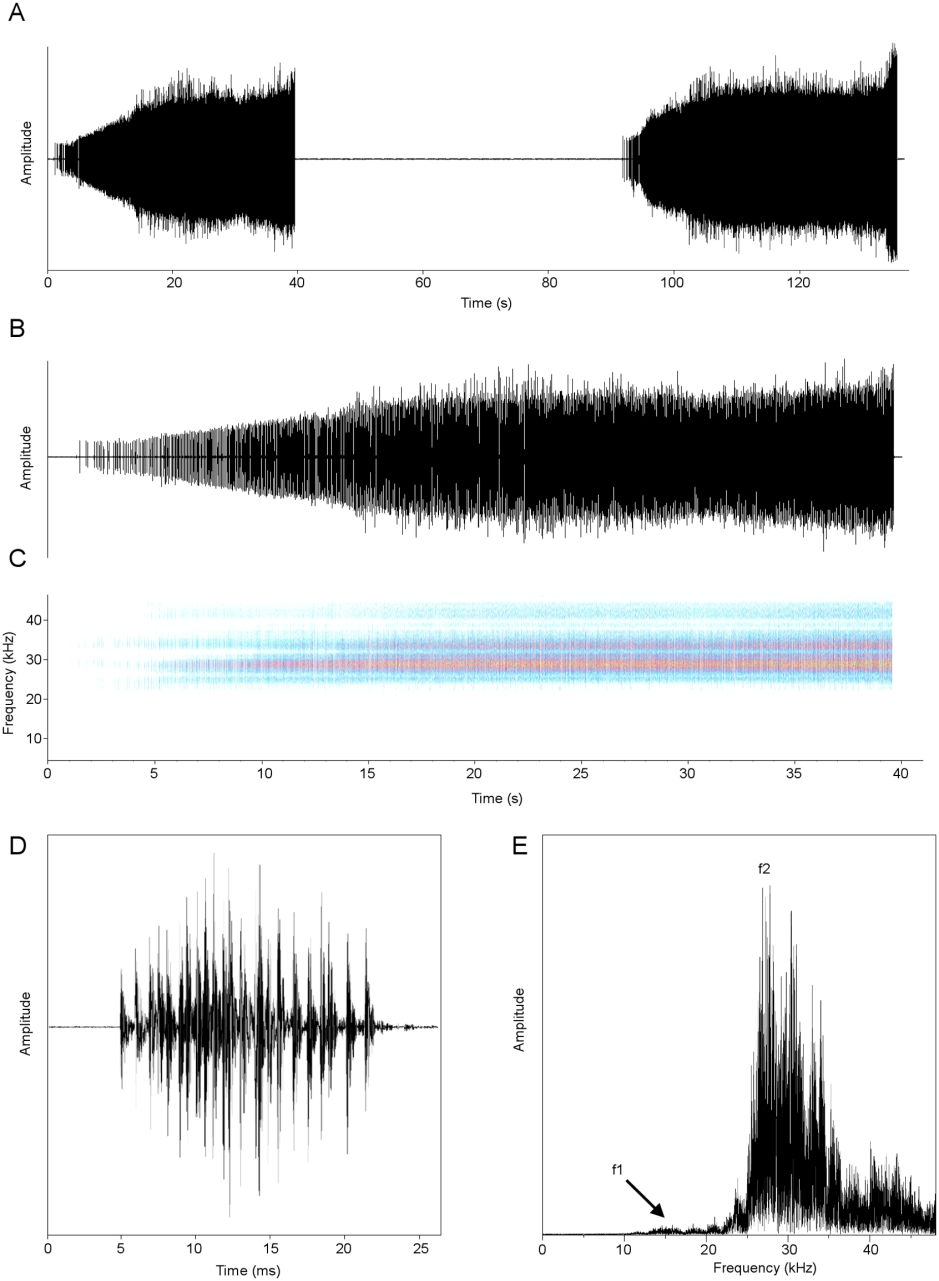
Calling song of *Pixibinthus sonicus*. (A) Oscillogram showing two successive trills; (B) detailed oscillogram and (C) sonogram of one trill; (D) oscillogram of one syllable; (E) and linear spectrogram of one echeme. Symbols: f1, fundamental frequency; f2, second harmonic (dominant frequency).

Four males were recorded under laboratory controlled condition. Each call of *Pixibinthus sonicus* is a trill lasting 38.2 ± 7.4 s (Fig 7A) and made of 918 ± 130 syllables, with a period of 152.8 ± 72.2 s (as recorded at 25°C). Syllables last18.4 ± 2.5 ms, with a period of 41.7 ± 16 ms, and with a duty cycle of 44.7%. The sound amplitude gradually increases during the 300 to 400 first syllables (Fig 7B), and remains twice higher and constant until the end of the trill. The dominant frequency is 27.9 ± 2.8 kHz, and corresponds to the second harmonic of the spectrum (Fig 7C and 7E), the fundamental frequency f1 being almost silent.

### Phylogenetic relationships of *Pixibinthus*

**Fig 8:**
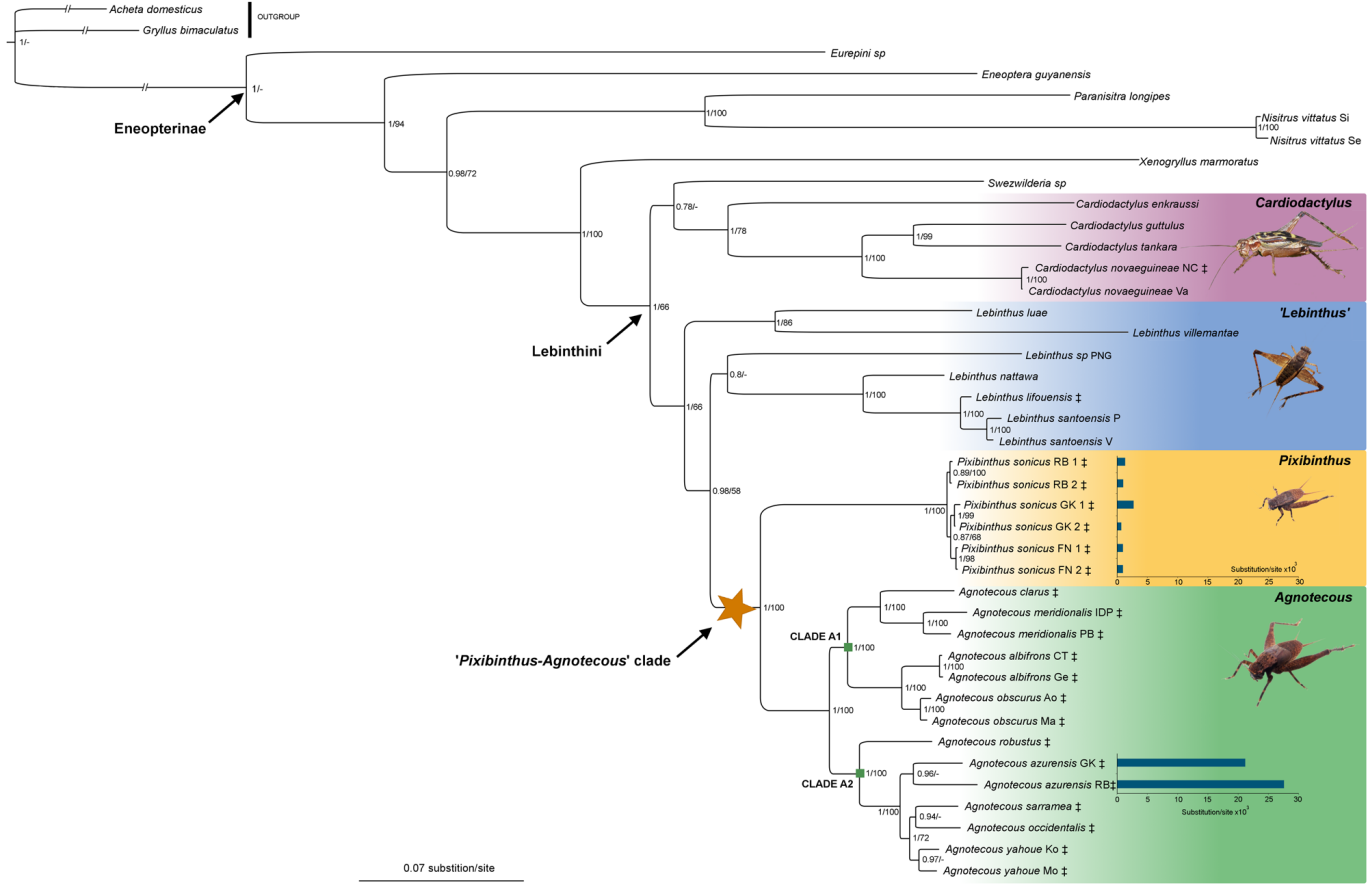
Bayesian majority rule consensus tree of Eneopterinae crickets based on dataset 1, including 28 species and nine DNA markers. Bayesian posterior probability (PP) andML bootstrap (BS) support values are indicated for each node on the right. A clade with a BS < 50% or not recovered in the ML analyses is indicated with a dash. Major clades are shaded with a color scale. Horizontal bars represent the scales of substitution rates (substitution per site). Species occurring in New Caledonia are represented with the ‡ symbol.

The ML and Bayesian MCMC single-locus phylogenetic topologies yield no supported incongruence for both datasets 1 and 2 (with a PP > 0.95 or BS > 85%; data not shown) and are less resolved than the combined phylogenetic analyses. The ML (data not shown), Bayesian MCMC and BEAST combined analyses provide no supported incongruence between topologies (Fig 8; S1 and S2 Figs). Since the Bayesian MCMC combined analysis allows us together to take into account uncertainty only the results of this analysis are discussed hereafter (Fig 8).

These analyses confirm that the genus *Pixibinthus* belongs to the Lebinthini tribe (PP = 1; Fig 8). The species *P*. *sonicus* is monophyletic, since all of the individuals sampled in this study group together with very short branch lengths (PP = 1, see below). Moreover *P*. *sonicus* is placed as sister to the monophyletic genus *Agnotecous* (PP = 1; Fig 8), and both form the newly-defined ‘*Pixibinthus*-*Agnotecous’* clade, with a high support (PP = 1). This latter lineage is nested with high support within the genus *Lebinthus* (PP = 0.98), a paraphyletic group under revision (Robillard et al. in prep). Within *Agnotecous* two main clades are recovered as sister groups. The *Agnotecous* clade A1 includes the *A*. *clarus*, *A*. *meridionalis*, *A*. *albifrons* and *A*. *obscurus* samples (PP = 0.99). The *Agnotecous* clade A2 comprises the *A*. *robustus*, *A*. *azurensis*, *A*. *sarramea*, *A*. *occidentalis*, and *A*. *yahoue* individuals (PP = 1).

The phylogenetic branch lengths (BL) estimated in this study are short in all *Pixibinthus* individuals (between 0.69 and 2.8 × 10^3^ substitutions/site; Fig 8), and larger in *Agnotecous* samples (mostly between 3.23 and 27.51 × 10^3^ substitutions/site, except *A*. *albifrons* with BL comprised between 1.38 and 1.69 × 10^3^ substitutions/site). Moreover the BL of *Pixibinthus* samples from the localities Grand Kaori and Rivière Bleue are 12 and 23 times shorter, respectively, than the BL of *A*.*azurensis* from the same places (compare *P*. *sonicus* RB and *P*. *sonicus* GK with *A*. *azurensis* RB and *A*. *azurensis* GK; Fig 8).

### Divergence time estimates

**Fig 9:**
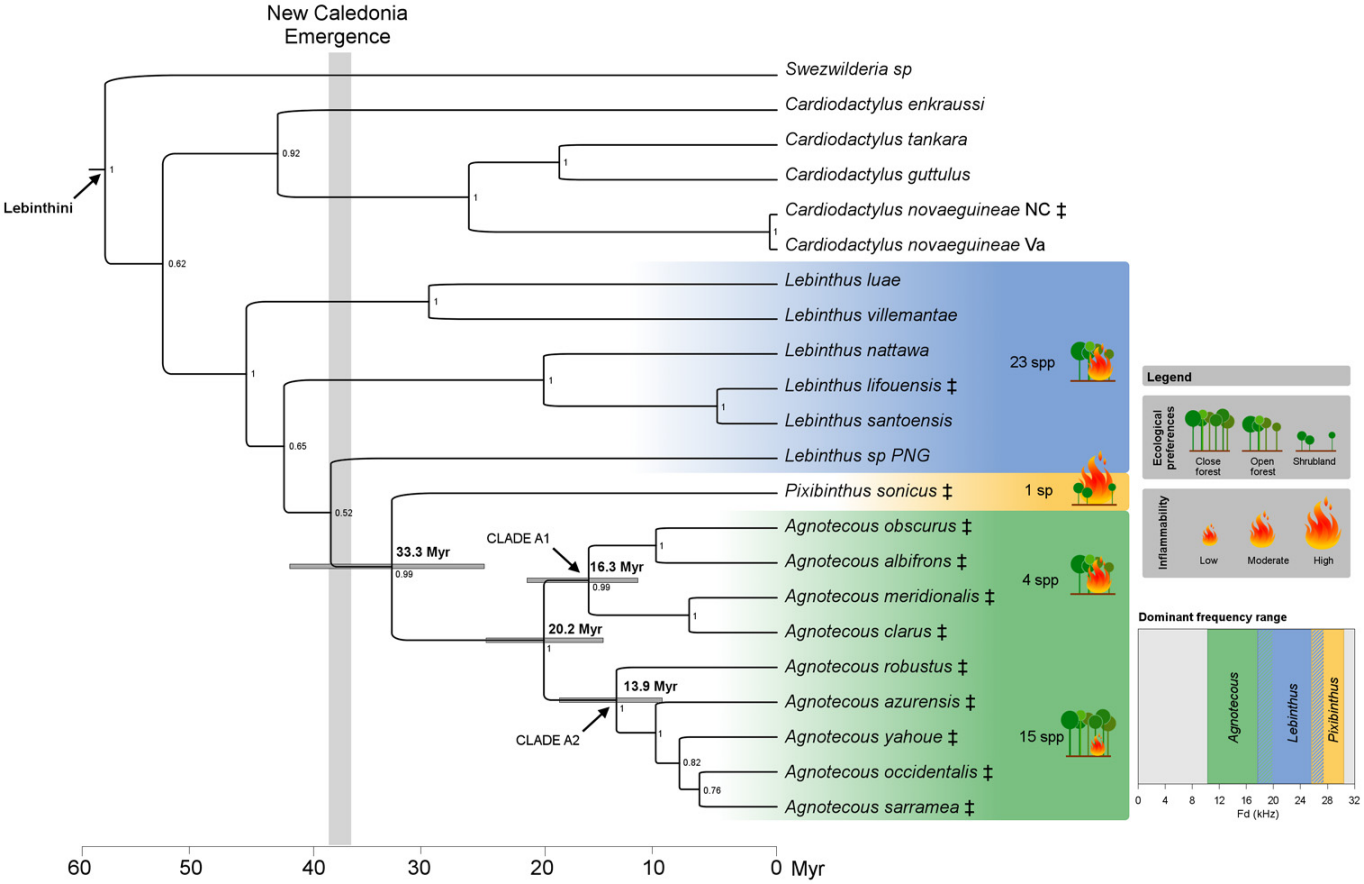
BEAST maximum credibility clade chronogram of the main lineages of Lebinthini crickets, based on dataset 2, including 41 species and seven DNA markers (only the 21 species of Lebinthini tribe are shown). Divergence times, ecological preferences and species richness (inferred from [26,61] and from our personal observations in the field), and relative habitat inflammability (inferred from [65])are indicated for the three main lineages: ‘*Lebinthus’*,*Pixibinthus*, and *Agnotecous*. Bayesian posterior probabilities (PP) are indicated for each node on the right. Gray node bars correspond to the 95% highest posterior density of median age estimates and are only provided for clades that are discussed. Dominant frequency range for the three mains lineages are indicated, with stripped area for overlapping frequency ranges. Species occurring in New Caledonia are represented with the ‡ symbol.

The BEAST analyses showed that the most recent common ancestor of the ‘*Pixibinthus*-*Agnotecous’* clade appeared around the Eocene-Oligocene boundary (with a median divergence time estimate of 33.3 Myr, with a 95% HPD of 24.4–43.1 Myr; Fig 9; Table 4). The crown ages of the genus *Agnotecous*, clade A1, and clade A2 were estimated around 20.2 Myr, 16.3 Myr, and 13.9 Myr, respectively (with 95% HPD of 14.9–25.82, 11.6–21.6, and 9.9–18.5 Myr, respectively; S2 Fig).

**Table 4:** Ecological, biological, morphological and dating characteristics for the three Lebinthini groups. Acoustic recording impaired by inadequate recording material are indicate with an asterisk. Body sizes of species were inferred from the FIIIL femur length measurements. Abbreviation: N/A corresponds to non-available data.

	*Pixibinthus*	*Agnotecous* clade A1	*Agnotecous* clade A2	*Agnotecous*	*'Lebinthus'*
*Characteristics*					
Number of species	1	4	15	19	23
Habitat	Shrubland, low to tall = 'maquis minier'	Secondary and open forest, savannah	Forest	Forest, secondary forest and savannah	Coastal and secondary forest, open forest
Habitat openness	Open	Open	Closed	Mainly closed to open	Open
Habitat inflammability	High	Moderate	Low	Mainly low to moderate	Moderate
Diets	Detritivorous	Detritivorous	Detritivorous	Detritivorous	Detritivorous
Dominant frequency range (kHz)	25.5–30.5	15–18	11–19.2	11–19.2	(12) 16.7–27.5
Body size (mm)	6.1–7.1	10.9–15.2	9.5–16.4	9.5–16.4	7–15.7
Substrate	Ultramafic	Ultramafic and Non-ultramafic	Ultramafic and Non-ultramafic	Ultramafic and Non-ultramafic	Non-ultramafic
*Age estimates*					
Stem age (Myr)	33.3	20.2	20.2	33.3	N/A
95% HPD stem age (Myr)	[24.4–43.1]	[14.9–25.8]	[14.9–25.8]	[24.4–43.1]	N/A
Crown age (Myr)	N/A	16.3	13.9	20.2	N/A
95% HPD crown age (Myr)	N/A	[11.6–21.6]	[9.9–18.5]	[14.9–25.8]	N/A

### Species richness estimates and relative diversification rates

The application of Equation (3) of Slowinsky and Guyer (1993) showed that *Pixibinthus* had significantly less species than its *Agnotecous* sister-clade (p = 0.0526*; one species *vs*. 19; Fig 8).

### Ensemble species distribution modelling

**Fig 10:**
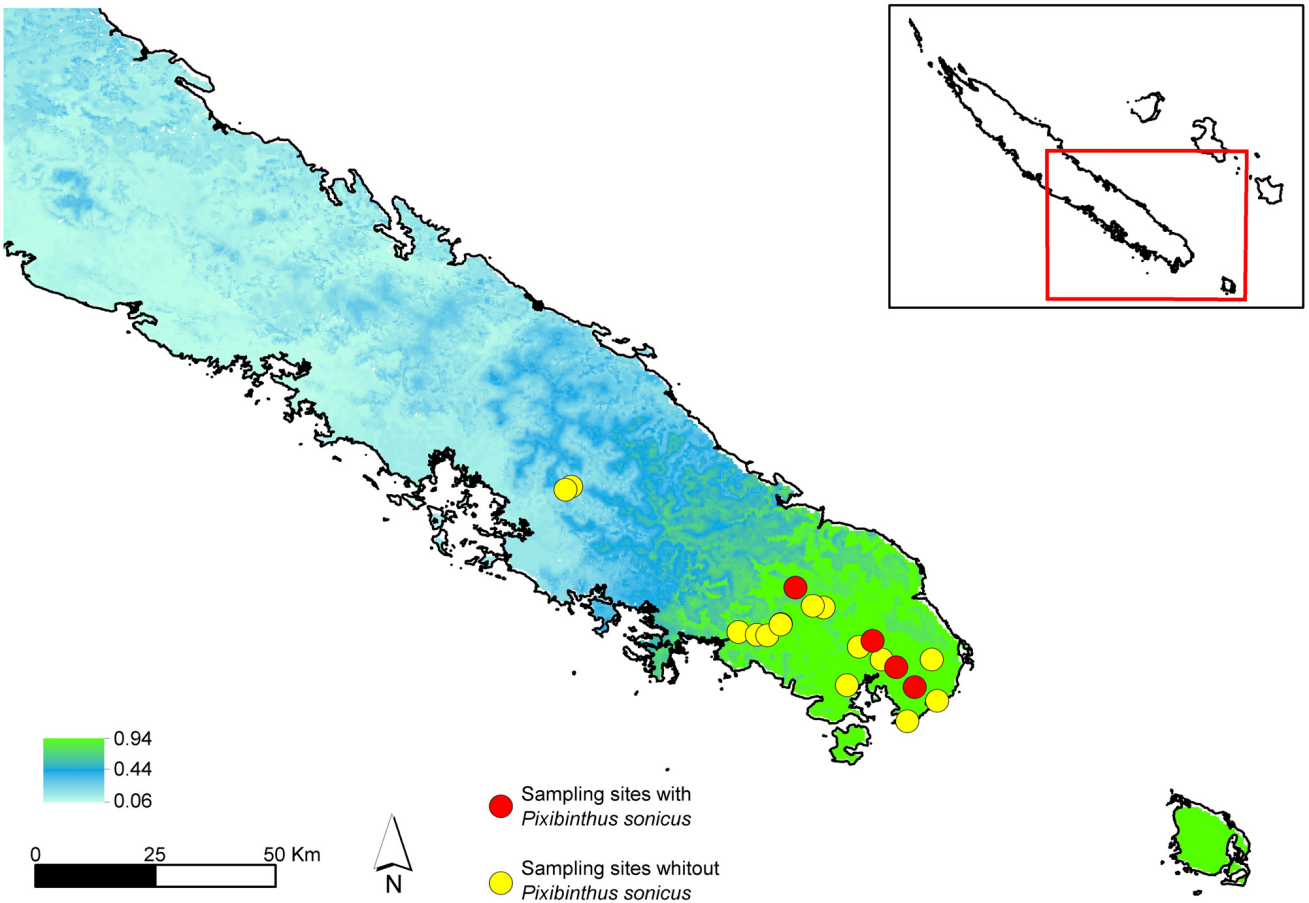
Predicted ecological distribution of *Pixibinthus sonicus* using ESDM. Warmer colors indicate areas with higher habitat suitability for *P*. *sonicus*. Reddots show sampling sites where *Pixibinthus sonicus* was recorded; yellowdots show sampling sites where *P*. *sonicus* was not observed.

ESDM analyses revealed that the suitable habitat of *Pixibinthussonicus* was not restricted to the four occurrence sites (AUC = 0.986), but also covered a large area of the southern of Grande Terre, exclusively on metalliferous soils (Fig 10).

## Discussion

### 1. The discovery of a new original cricket genus in ‘maquis minier’

In this study we described a new monotypic cricket genus, *Pixibinthus*, endemic to New Caledonia. Its single species *P*. *sonicus*, has only been observed in the southern part of New Caledonian main island, living in specific open shrubby vegetation on ultramafic rocks (i.e. ‘maquis minier’; Table 4). Unexpectedly, *P*. *sonicus* exhibits the highest calling frequency ever recorded for any Grylloidea species, a record previously heldby *Lebinthus santoensis* at 26.5 kHz in Vanuatu [66]. Our phylogenetic study placed *Pixibinthus* as sister to the genus *Agnotecous*, also endemic to Grande Terre (Fig 8). This study confirms that the genus *Lebinthus* is paraphyletic as suggested by recent taxonomic [30] and phylogenetic studies [27,40].

### 2. Origin of the ‘*Pixibinthus-Agnotecous*’ clade in New Caledonia

The origin of the ‘*Pixibinthus-Agnotecous’* clade in New Caledonia was estimated in our study to ca. 33.3 Myr (with a 95% HPD of 24.4–43.1 Myr; Fig 9; Table 4). Before the discovery of the close relationship between these two genera, the early colonization of New Caledonia by *Agnotecous* was previously estimated between 10.6 and 15.3 Myr (with a 95% HPD of 5.3–21.9 Myr; [40]). The inclusion of *Pixibinthus* in our DNA sampling also helped refining the age estimate of *Agnotecous* in New Caledonia. Our dating results pushed back the crown age of the *‘Pixibinthus-Agnotecous* clade’ up to ca. 23 Myr. [40] also tentatively incorporated an extreme fossil calibration using a very old but dubious fossil of Grylloidea in order to test some specific biogeographical hypotheses. Their second analysis dated *Agnotecous* at approximately 24 Myr, which already permitted to reject the assumption of a clade older than the island. However this result was not retained as a robust estimate within that study, given the uncertainty on the fossil used for calibration. Strikingly our results however gave very similar estimates, despite the differences in dating methods, calibrations, and taxonomic and gene samplings.

According to our results, the ‘*Pixibinthus-Agnotecous* clade’is an old New Caledonian lineage that arrived not long after the postulated re-emergence of New Caledonia, estimated by geologists around 37 ± 2 Myr [6,7], similarly to some other groups of terrestrial arthropods such as fig wasps [67] or caddisflies [68]. Although *Pixibinthus* presently appears as a relict, actually a unique taxon on a long phylogenetic branch [69], its origin does not seem to predate the emergence of the island as for some harvestmen [70] tentatively assumed to have jumped over now-drowned islands earlier than 37 My ago [40]. This provisional relict status depends on further sampling that could reveal other extant species belonging to the same genus, though several and extensive previous studies focusing on forest habitats did not find any species [25,26,27].

According to classical accounts on New Caledonia, old clades should primarily occupy habitats on metalliferous soils which were dominant at the time of emergence of the island [10,12,71,72]. The relationship between *Pixibinthus* and ‘maquis minier’ is consistent with this assumption, though its sister-group *Agnotecous* is not restricted to metalliferous soils [27].

The common ancestor of this cricket clade probably settled in early New Caledonian open habitats, a preference which can be inferred as ancestral for this lineage, also considering the preference for open forests of close relatives such as Indo-Pacific *Lebinthus* species [28,66,73] (Fig 9). A similar pattern has already been observed in *Cardiodactylus novaeguineae*, whose ongoing colonization is documented in open backshore habitats of New Guinean and Melanesian archipelagoes, including New Caledonia [74]. New Caledonia would then have been successively colonized three times by crickets of the subfamily Eneopterinae: the ‘*Pixibinthus-Agnotecous* clade’, *C*. *novaeguineae*, which has a wide distribution in the Western Pacific, and *L*. *lifouensis*, restricted to the Loyalty Islands ([26,40]; Fig 8).

### 3. Two different ecological specializations within the ‘*Pixibinthus-Agnotecous*’ clade

Interestingly, the *Pixibinthus* lineage would have persisted in the seemingly ancestral choice of open habitats since its origin in New Caledonia. This species appears to have specialized upon the emblematic and peculiar sclerophyllous shrubby‘maquis minier’ growing on ultramafic rocks ([11,75]; Table 4). The exact timing of this specialization could not be inferred in this study, but only estimated between around 33 Myr to present, as this species is the only representative of the genus.

The sister-group *Agnotecous* differs, as 16 out of its 19 species are rainforest-dwellers (*Agnotecous* clade A2 plus *A*. *meridionalis*), while only three other species (clade A1) are known from more open forests ([26,27,61]; Table 4; S2 Table). According to the topology, occurring in closed habitatsis however a secondary specialization within *Agnotecous*(clade A2: 13.9 Myr; Fig 9). We can confidently hypothesize that the postulated radiation of *Agnotecous* in forest habitats started around 20 Myr (crown age of the genus *Agnotecous*; Table 4).

### 4. Ecological specializations suggest mutual evolution of body size and airborne signal

To the best of our knowledge, the clear-cut ecological specializations in two vegetation types of two sister-clades (*Agnotecous* mostly in closed forest and *Pixibinthus* in open ‘maquis minier’; Table 4) is undocumented within the New Caledonian insect fauna. In this unusual case, the study of acoustic behavior could be a relevant feature to understand such different ecological preferences.

Before the discovery of *Pixibinthus*, [76]described the few Lebinthini as ‘living paradoxes’, since they were producing high-frequency calls in forested habitats (S2 Table), which are characterized by greater attenuation and absorption on those frequencies [77,78]. But now, the significantly lower frequency calls observed in *Agnotecous* species (compared to *Pixibinthus*) might be explained by new acoustic and ecological constraints inherent to forest habitats (Fig 9, dominant frequency range diagram). The colonization of forested areas would have allowed more favorable living conditions for the ancestor of *Agnotecous*, with a release of ecological pressures. Resources, including food and shelter from predators, were presumably greater than in open habitats [79,80], which is usually associated with an increase of body size([81,82]; Table 4). Consequently, the *Agnotecous* lineage would have undergone a relative reduction of their call frequencies as a consequence to a larger body size [83], whereas *Pixibinthus* would have conserved a smaller body size and a high-frequency song (S2 Table). A formal test of this hypothesis will necessitate further studies taking into account precise acoustic analyses at the scale of whole clade analyzed with phylogenetic comparative methods, especially to identify the role of body size and habitat characteristics on the evolution of call frequencies (e.g. [84]).

In crickets, calling songs are crucial to attract females, and are strongly influenced by environmental conditions [85]. *Pixibinthus sonicus* exhibits a high-frequency call compared to *Agnotecous*, and this species lives in open habitats, where sound transmission is strikingly different than in forest areas: amplitude fluctuations are higher and reverberation is lower, which tends to facilitate high-frequency communication [78,86,87]. In addition, the open ‘maquis minier’ where *P*. *sonicus* lives is usually composed of patchy ‘leaf litter islands’, where conspecific potential receivers are likely to be concentrated in the close vicinity of male signalers (as according to our field observations). These circumstances may have concurred to maintain, or favor, high-frequency communication in *Pixibinthus*.

### 5. *Pixibinthus*: failing to diversify?

Despite a large temporal window, we observed imbalanced species richness between *Pixibinthus* and *Agnotecous* (as shown by a significant p-value of the Slowinsky and Gruyer test). We noticed also important dissimilarities in species richness with a single species for *Pixibinthus* vs. 19 for *Agnotecous* (Table 4). Both genera are detritivorous, as most crickets, meaning that diet might not explain this difference. Furthermore, although the knowledge on their predation pressure and biotic interactions is scarce, similar categories of potential predators seem present in both environments (mainly spiders and lizards). Based on that statement, we propose a working hypothesis to explain these uneven diversifications considering past and recent abiotic events, relative to climatic oscillations and fire regimes.

#### 5.1. Impact of past climatic fluctuations?

Climatic fluctuations are greatly suspected to have been an important factor responsible for the natural expansion and contraction of New Caledonian rainforests over time [15,88,89]. As an example, allopatric speciation has been documented as one of the main diversification driver for the rainforest-dwelling *Agnotecous* [27]. Past expansion-contractions of this vegetation have been shown in southern main land through palynological records [65,90,91]. If such weather shifts had been critical in open shrubby habitats, leading to the isolation of ‘maquis minier’ patches, we should observe as many allopatric speciations in *Pixibinthus* as in *Agnotecous* a situation that is not supported by our data. On the contrary, the southern plain ‘maquier miniers’ could have been permanently connected [90,92]. *Pixibinthus* may then have failed to diversify because of relatively high habitat connectivity, regardless to its dispersal ability. The gene flux between distant populations could have been maintained over time, limiting allopatric speciation, as suggested by short internal phylogenetic branches within *P*. *sonicus*. The contrast with some *Agnotecous* species, whose isolated forested populations begin to be genetically isolated (such as illustrated by long branches of *A*. *azurensis*; Fig 8), could then be particularly significant.

#### 5.2. Impact of fire regimes?

Fire regime may have equally played a key role in the apparent lack of diversification of *Pixibinthus*. Before human arrival (ca. 3000 year ago), New Caledonian landscapes were shaped by low natural fire regime [21,90]. As the exclusive ecological preference of *Pixibinthus* matches the most flammable habitat of New Caledonia [65], which are now exposed to repeated fires [93], could have led to occasional and localized extinctions, enhancing isolation of its populations in a first phase. However, these newly burnt areas would in time have constituted a favorable matrix for recolonization, ultimately leading to population reconnections. By contrast, fire damages are expected to be buffered by dense forested areas [21,94]. The specialization of *Agnotecous* in forest habitats would have preserved its populations, allowing the emergence of many species according to the hypothesis proposed by Nattier et al. [27]. In this likely scenario, we might assume that *Pixibinthus* would be more sensitive to fire damages than *Agnotecous*.

### 6. *Pixibinthus*: on the way to extinction?

More recently, fire regime was dramatically increased by human’s arrival in New Caledonia, as documented in the southern region by palynological and charcoal records from Quaternary sediments [65,90,91]. According to McCoy et al. [65], fire frequency has increased from one fire to five every century since human settlement. This recent (less than 3000 years) and abnormally high fire regime would have increased the effect of natural fire on native vegetation, and especially in shrubby ‘maquis minier’. Though, repeated occurrence of dramatic fire events would have led to the loss of 10.000 ha of vegetation per year in New Caledonia, of which 10% in the southern part (civilian security of New Caledonia, pers. comm.). These recent human-made fires are mostly sudden and destroy very large areas in short time. For example, 4300 and 800 ha were burnt in 2005 and 2013, respectively (civilian security of New Caledonia, pers. comm.). This increasing change in fire regime created a large vegetation mosaic of maquis-forest, dominated by flammable maquis, especially in the southern part of New Caledonia. This mosaic influence fire regime with more maquis prone to burn rather than forest remnants [93,95]. Before man arrival, the low regime of fire was related to a maquis-forest mosaic dominated by forest, which we hypothesize offer more opportunity of stable open shrubby maquis areas available for *Pixibinthus* population with a low turn over (a mosaic of maquis minier of differents ages in a matrix of forest). According to the actual fire regime, Curt et al. [93] estimate that more than 10 000 ha of maquis have burnt over the period 1999–2010. These authors also estimate that with a such fire regime it would take 34 years to burn an area equivalent to the whole area of maquis in New Caledonia. The known distribution of *Pixibinthus* being ca. 1.200 ha (Fig 5), means that its geographical range could have been virtually entirely burnt within one year. Furthermore, since the potential sustainable habitat of *Pixibinthus* is larger than its actual distribution, as inferred by both niche modelling analyses (Fig 10), the geographical expansion of this cricket lineage could have been held back by environmental perturbations, like drastic fire regimes, rather than by limited living conditions. As a consequence, the probability of extinction of *Pixibinthus* populations may have been greatly enhanced by human arrival, preventing recolonization processes and reconnection between isolated populations, even if the favorable habitat of this *Pixibinthus* is considerably enlarged.

### 7. Conclusion

Our study highlights an original discovery for the cricket fauna in emblematic south-eastern ‘maquis miniers’ from New Caledonia. The new endemic monotypic genus *Pixibinthus* appears to have originated from an old lineage contemporary to the 37 Myr-re-emergence of the main island. Its single species would have conserved ancestral traits for open habitats with high-frequency calls (up to 30 kHz). This lineage could be the result of a tumultuous history involving two mutually non-exclusive evolutionary patterns: (1) an apparent non-speciation linked to the past dynamics of its particular habitat, and (2) extinctions caused by a recent high human-made fire regime. Nevertheless, the long-branch phylogenetic signature of the new genus is difficult to interpret, which could be elucidated by a relevant genetic population study on the entire distribution range of this species. So far, the scarceness and the observed distribution of the species, restricted to the south raises issues about its presence in the other parts of New Caledonia. Additional sampling efforts should be done in other localities, well distant from the current known distribution, as for example in ‘maquis minier’ located at high-elevations or those located in north-western isolated mountains, on which allopatric speciation events have occurred in plants [96,97], geckos [98,99], skinks [100,101] and grasshoppers [16]. In New Caledonia clear-cut specializations between two sister lineages have been frequently highlighted for different geological substrates in insects (e.g. in caddisflies [14], or in grasshopper [16,102]), in squamata [99,100,101] or in plants (e.g. in *Codia sp*. [103], or *Diospyros sp*. [13]). However ecological specialization in contrasted vegetation types, such as shown in our study, was undocumented for New Caledonia, even if it is strongly suspected in some insect groups (e.g. in some Chrysomelid beetles or weevil beetles, in some Mogoplistidae lineages, or in the *Bullita*-*Koghiella* clade) or plant groups (e.g. in the *Morierina*–*Thiolliera* clade or within the large *Psychotria*). Our findings stressed the importance to study animal or plant diversifications by considering all stages of ecological succession, such as disturbed habitat (e.g. shrubby vegetation), which could provide valuable information to understand the origins of New Caledonian biodiversity, massively threatened by anthropogenic pressures.

## Supporting Information

S1 Fig Bayesian majority ruleconsensus tree of Grylloidea crickets based on dataset 2, including 41 species and seven DNA markers. Bayesian posterior probability (PP) / ML bootstrap (BS) support values are indicated for each node on the right. A clade with a BS < 50% or not recovered in the ML analyses is indicated with a dash.
(TIF)

S2 Fig BEAST maximum credibility clade chronogram of Grylloidea crickets based on dataset 2, including 41 species and seven DNA markers. Bayesian posterior probability (PP) / bootstrap (BS) support values are indicated for each node on the right. A clade with a BS < 50% or not recovered in the ML analyses is indicated with a dash. Gray node bars correspond to the 95% highest posterior density of median age estimates.
(TIF)

S1 Table Characteristics of the nine DNA loci used in this study and in the phylogenetic analyses of *Pixibinthus* and closely related genera. A) Characteristics of primers (names, sequences, annealing temperatures, references where they were first designed). Characteristics of the DNA partitions, used in the phylogenetic analyses: B) ‘dataset 1’: for phylogenetic relationships; C) ‘dataset 2’: for dating estimates.
(XLS)

S2 Table Preferential habitat and dominant frequency range of *Lebinthus* (only species included in the molecular study), *Agnotecous* and *Pixibinthus* species. Acoustic recording obtained with inadequate recording material are indicated with an asterisk. Unpublished data are indicated with two asterisks. Species only known from the type specimen are indicated with three asterisks. Abbreviation: N/A corresponds to unavailable data.
(XLSX)

S3 Table List of localites for the cricket sampling with geographical coordinates. (XLSX)
