# Supplementary figures and images for: Old Lineage on an Old Island: Pixibinthus, a New Cricket Genus Endemic to New Caledonia Shed Light on Gryllid Diversification in a Hotspot of Biodiversity
Source: PLoS One. 2016 Mar 30;11(3):e0150920. doi: 10.1371/journal.pone.0150920 (PMC4814057; doi:10.1371/journal.pone.0150920)

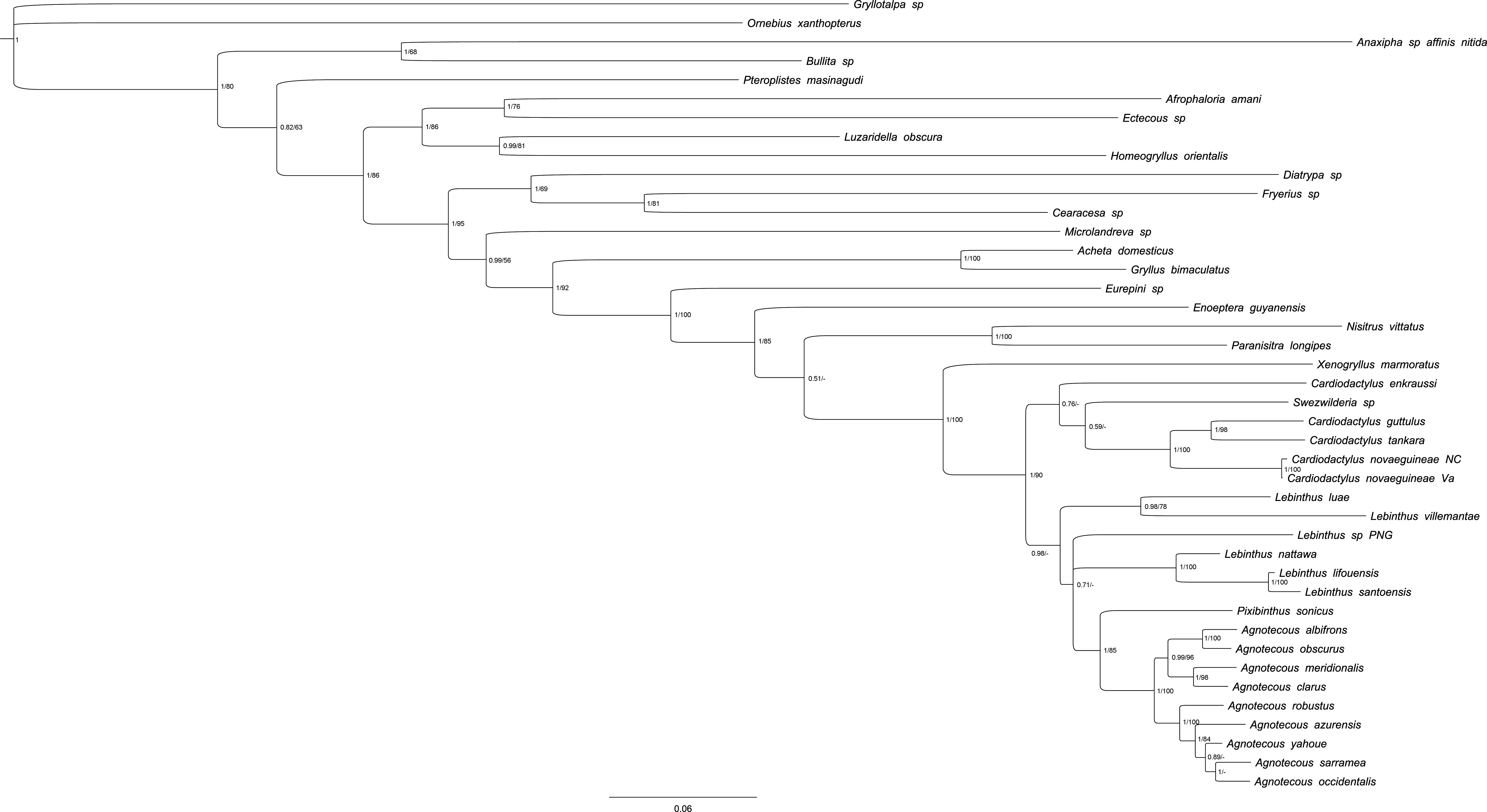

Supplement: S1 Fig — Bayesian posterior probability (PP) / ML bootstrap (BS) support values are indicated for each node on the right. A clade with a BS < 50% or not recovered in the ML analyses is indicated with a dash. (TIF) [file pone.0150920.s001.tif]

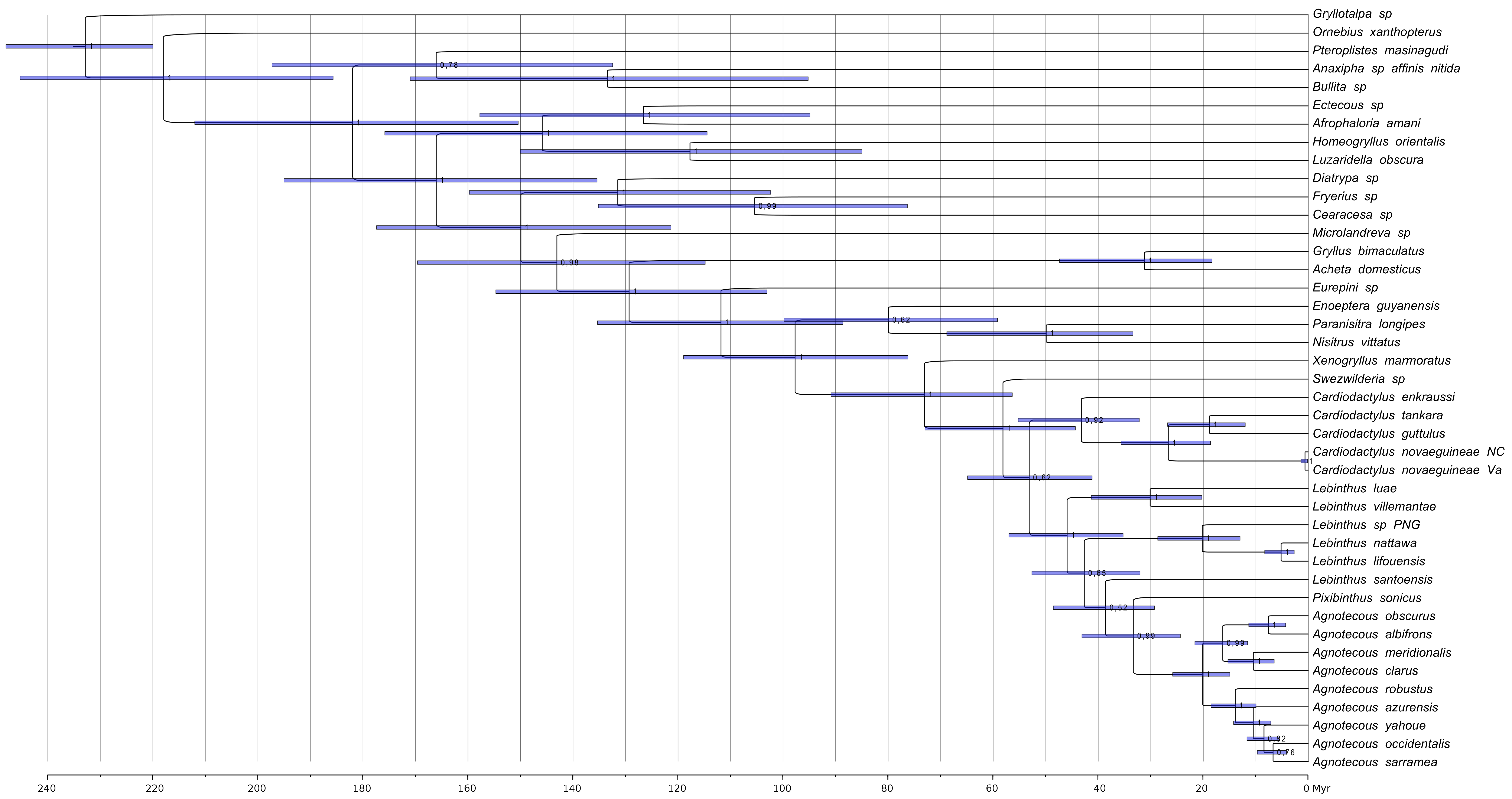

Supplement: S2 Fig — Bayesian posterior probability (PP) / bootstrap (BS) support values are indicated for each node on the right. A clade with a BS < 50% or not recovered in the ML analyses is indicated with a dash. Gray node bars correspond to the 95% highest posterior density of median age estimates. (TIF) [file pone.0150920.s002.tif]
